# Bismuth Chalcogenide‐Based Photocatalysts for Nitrogen Reduction: Recent Progress and Prospects

**DOI:** 10.1002/chem.71414

**Published:** 2026-07-15

**Authors:** Mukesh K. Verma, Muhammad D. Bala, Phindile B. Khoza, Tshephiso R. Papo, Mzamo L. Shozi

**Affiliations:** ^1^ Discipline of Chemistry School of Agriculture and Science University of KwaZulu‐Natal Durban South Africa

**Keywords:** ammonia synthesis, bismuth chalcogenides, defect engineering, photocatalytic nitrogen reduction

## Abstract

Bismuth chalcogenides and related bismuth‐based materials have recently attracted considerable attention as promising photocatalysts for sustainable nitrogen reduction reaction (NRR) due to their unique electronic structures, visible‐light responsiveness, stereochemically active Bi 6s^2^ lone pair, and tunable band positions that facilitate nitrogen adsorption and activation. This review summarizes recent advances in the rational design of bismuth chalcogenide‐based photocatalysts for light‐driven ammonia synthesis. Fundamental aspects of photocatalytic N_2_ reduction and reliable methodologies for ammonia quantification are critically discussed. Material engineering strategies, including defect modulation, elemental doping, heterojunction construction (Type‐II, Z‐scheme, and S‐scheme systems), and hybrid structural design, are analyzed with respect to charge separation and band alignment. Key challenges, such as low N_2_ activation efficiency, competing hydrogen evolution reaction, charge recombination, and reproducibility concerns, are highlighted. Future perspectives emphasize electronic structure regulation, operando mechanistic studies, standardized evaluation protocols, and integration with photoelectrochemical systems for improved efficiency and selectivity.

## Graphical abstract

Bismuth chalcogenides emerge as promising visible‐light photocatalysts for sustainable ammonia synthesis, driven by tunable electronic structures and strong N_2_ activation. Recent advances reveal how defects, doping and heterojunction design synergistically enhance charge separation and reactivity. Key challenges and future directions highlight pathways toward efficient, reproducible and scalable solar‐driven nitrogen fixation systems.

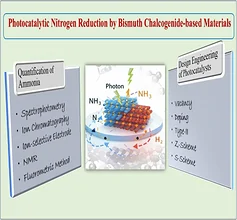

## 1 Introduction

Rapid industrialization and modernization have led to a significant increase in global energy demand and environmental deterioration, making these challenges central to modern society. The growing energy consumption has intensified greenhouse gas emissions and aggravated the global energy crisis, posing serious risks to human health and ecological stability [1]. Currently, the majority of energy production relies on finite, nonrenewable fossil fuels such as coal, oil, and natural gas. The continued reliance on these resources results in substantial carbon dioxide emissions, as well as harmful pollutants such as carbon monoxide, nitrogen oxides, and sulfur oxides. Encouragingly, the expansion of renewable energy sources contributed to an approximate 7% reduction in fossil fuel consumption in 2018 [2]. Among the various alternatives, solar energy conversion has become a particularly promising approach for reducing greenhouse gas emissions and advancing sustainable development [3].

Ammonia (NH_3_) is an essential industrial chemical widely used in the manufacture of commercial and household products, particularly fertilizers and cleaning agents. In addition, it plays key roles as a refrigerant, stabilizer, neutralizing agent, and purifier in processes such as food preservation and water treatment. Ammonia is also employed in the production of explosives, plastics, dyes, textiles, and pharmaceuticals [4]. Beyond its conventional applications, ammonia has gained increasing attention as a potential hydrogen (H_2_) carrier due to its high energy density, ease of liquefaction, and capability to release hydrogen upon catalytic decomposition, making it a promising candidate for energy storage and transport [5]. For this reason, NH_3_ is considered for sustainable energy systems.

Currently, industrial ammonia production is dominated by the Haber–Bosch process. Although this process revolutionized global fertilizer production and supported modern agriculture, it operates under energy‐intensive conditions, typically requiring temperatures of 400°C–500°C and pressures of 150–300 bar [6]. Such harsh operating parameters lead to considerable energy demand, accounting for nearly 2% of global energy use, and contributing approximately 1.44% of annual CO_2_ emissions worldwide [7, 8]. The heavy reliance on fossil‐derived hydrogen sources, including natural gas, oil, and coal, further amplifies carbon emissions and environmental concerns. Given the escalating challenges of climate change, fossil fuel depletion, and environmental degradation, it is necessary to develop sustainable, energy‐efficient alternatives to ammonia synthesis under mild conditions. In this context, ammonia production routes, particularly those driven by renewable energy, have emerged as promising strategies to reduce carbon footprints and promote long‐term environmental sustainability.

Nanomaterials have attracted significant attention in energy conversion and environmental remediation owing to their unique physicochemical properties, including high surface area, tunable electronic structures, and enhanced catalytic activity [9]. Upon light irradiation, semiconductor materials generate electron–hole pairs that can interact with water and dissolved oxygen to form reactive oxygen species (ROS). These reactive species play crucial roles in various photocatalytic processes, such as nitrogen reduction (NRR), carbon dioxide reduction, hydrogen peroxide production, solar‐driven water splitting, and the degradation of organic pollutants [10, 11]. Notably, semiconductor‐based photocatalysts and photo‐electrocatalysts operate under mild conditions, typically at room temperature and ambient pressure, while utilizing renewable energy sources such as sunlight. This sustainable approach not only enhances catalytic efficiency but also helps mitigate energy shortages and environmental pollution. In this context, the photocatalytic conversion of nitrogen and water into ammonia (NH_3_) has emerged as a promising and environmentally benign alternative to the energy‐intensive Haber–Bosch process [12]. The development of efficient semiconductor photocatalysts capable of activating and reducing inert N_2_ under ambient conditions has become a central focus of research. In some previous work, TiO_2_ [13], g‐C_3_N_4_ [14], Sn‐doped MOF [15], Fe‐doped TiO_2_ [16], and N‐doped porous carbon [17] photocatalysts were used for the photocatalytic and electrocatalytic reduction of molecular nitrogen to ammonia. Among various candidates, bismuth‐based materials have emerged as promising systems for photocatalytic nitrogen reduction, as they are nontoxic, noncarcinogenic, and exhibit excellent photocatalytic activity across many fields. Their unique electronic structure, long Fermi wavelength, suitable band gap (typically in the visible‐light region) and surface plasmon resonance effect enable effective solar light absorption and facilitate charge‐carrier migration. More importantly, the presence of Bi 6p orbitals enables favorable orbital interactions and potential π back‐donation into the antibonding orbitals of N_2_, thereby weakening the N≡N bond and promoting its activation [8, 18, 19].

Despite these intrinsic advantages, pristine bismuth chalcogenides such as Bi_2_O_3_, Bi_2_S_3_, Bi_2_Se_3_, and Bi_2_Te_3_ often exhibit limited nitrogen adsorption capacity, rapid electron‐hole recombination, and suboptimal band‐edge alignment for ammonia formation [20, 21]. To address these challenges, various strategies, including defect engineering, elemental doping, heterojunction construction, and morphology control, have been employed to enhance charge separation, tune electronic structures, and increase the number of active sites [22]. These advances have significantly improved the efficiency of photocatalytic nitrogen fixation and positioned bismuth chalcogenides and their derivatives as an important and rapidly evolving class of materials for sustainable ammonia synthesis. In this review, we first discuss the key parameters for evaluating photocatalytic nitrogen reduction performance, with particular emphasis on reliable methods for ammonia quantification, including spectrophotometry, ion chromatography (IC), ion‐selective electrode analysis, NMR techniques, and fluorometric detection. Subsequently, we provide a comprehensive overview of photocatalytic bismuth chalcogenides for nitrogen reduction reaction (NRR), focusing on defect‐engineered systems (vacancy creation and doping strategies), heterojunction architectures (Type‐II, Z‐scheme, and S‐scheme systems), and bismuth chalcogenides integrated into porous or hybrid host matrices. Finally, the remaining challenges and future research perspectives are outlined to guide the rational design of highly efficient bismuth‐based photocatalysts.

## 2 Fundamentals of Photocatalytic N_2_ Reduction

The photocatalytic reduction process is initiated when the photocatalyst absorbs photons with energies equal to or greater than its band gap, thereby promoting electrons from the valence band (VB) to the conduction band (CB). The resulting photogenerated electrons and holes migrate to the active sites, where they drive various surface reactions, including pollutant degradation, hydrogen evolution, oxygen reduction or evolution, and nitrogen reduction. The overall photocatalytic performance depends on the light‐harvesting efficiency, charge separation and migration, electron–hole recombination rate, and the reactant's molecular properties, including its structure, bond energy, polarity, redox potential, and adsorption affinity toward the catalyst surface [23].

Nitrogen is an inert molecule with a high ionization energy (15 eV), negative electron affinity (−1.8 eV), large bond dissociation energy (941 kJ mol^−1^), absence of a permanent dipole moment, and a wide band gap (22.9 eV), making its activation highly challenging [24, 25]. The heterogeneous catalytic reduction of N_2_ proceeds via distinct pathways. In the dissociative mechanism, the N─N bonds are first cleaved, which is an energy‐intensive step, followed by stepwise hydrogenation. Owing to its high energy requirements, this pathway is uncommon in photocatalysis but forms the basis of the Haber–Bosch process, which requires high temperatures and pressures [26]. In contrast, the associative mechanism proceeds through three possible pathways: alternating, distal, and enzymatic, as illustrated in Figure 1. In the alternating and distal pathways, N_2_ molecules adsorb perpendicularly to the catalyst surface, followed by a stepwise hydrogenation. In the alternating pathway, hydrogenation occurs alternately on both nitrogen atoms, whereas in the distal pathway, the terminal (distal) nitrogen atom is fully hydrogenated first to form one NH_3_ molecule, followed by hydrogenation of the remaining nitrogen atom to yield the second NH_3_ molecule. In the associative alternating pathway, partially hydrogenated nitrogen species such as diazene (N_2_H_2_*) and hydrazine (N_2_H_4_*) can form as key intermediates, which may also appear as undesired byproducts under certain reaction conditions. Upon further hydrogenation, these intermediates can ultimately be converted into ammonia (NH_3_). In particular, the detection or possible formation of N_2_H_4_ is often considered evidence for the operation of the alternating mechanism during the hydrogenation process [27, 28]. The required protons and electrons for hydrogenation are supplied by the reaction medium and the active catalyst. Unlike the dissociative mechanism, these associative pathways avoid the initial cleavage of the N≡N bond, thereby requiring significantly lower activation energy. Consequently, photocatalytic and electrocatalytic NRRs predominantly proceed via the associative mechanism [29, 30]. In the associative enzymatic nitrogen reduction process, both nitrogen atoms of the N_2_ molecules are simultaneously adsorbed on the catalyst, followed by successive hydrogenation of both atoms until two ammonia molecules are generated, desorbed and released from the enzyme surface [31]. The Mars‐van Krevelen (MvK) pathway has been identified as a new candidate for electrocatalysis of transition metal nitrides (TMNs). In this process, the initial ammonia molecules are formed through the reduction of nitrogen atoms embedded within the TMN lattice. These nitrogen sites are subsequently replenished by the incorporation of gaseous dinitrogen. To confirm the operation of this mechanism in electrocatalytic systems, researchers often employ ^15^N isotope labeling experiments as a tracing method [32].

**FIGURE 1:**
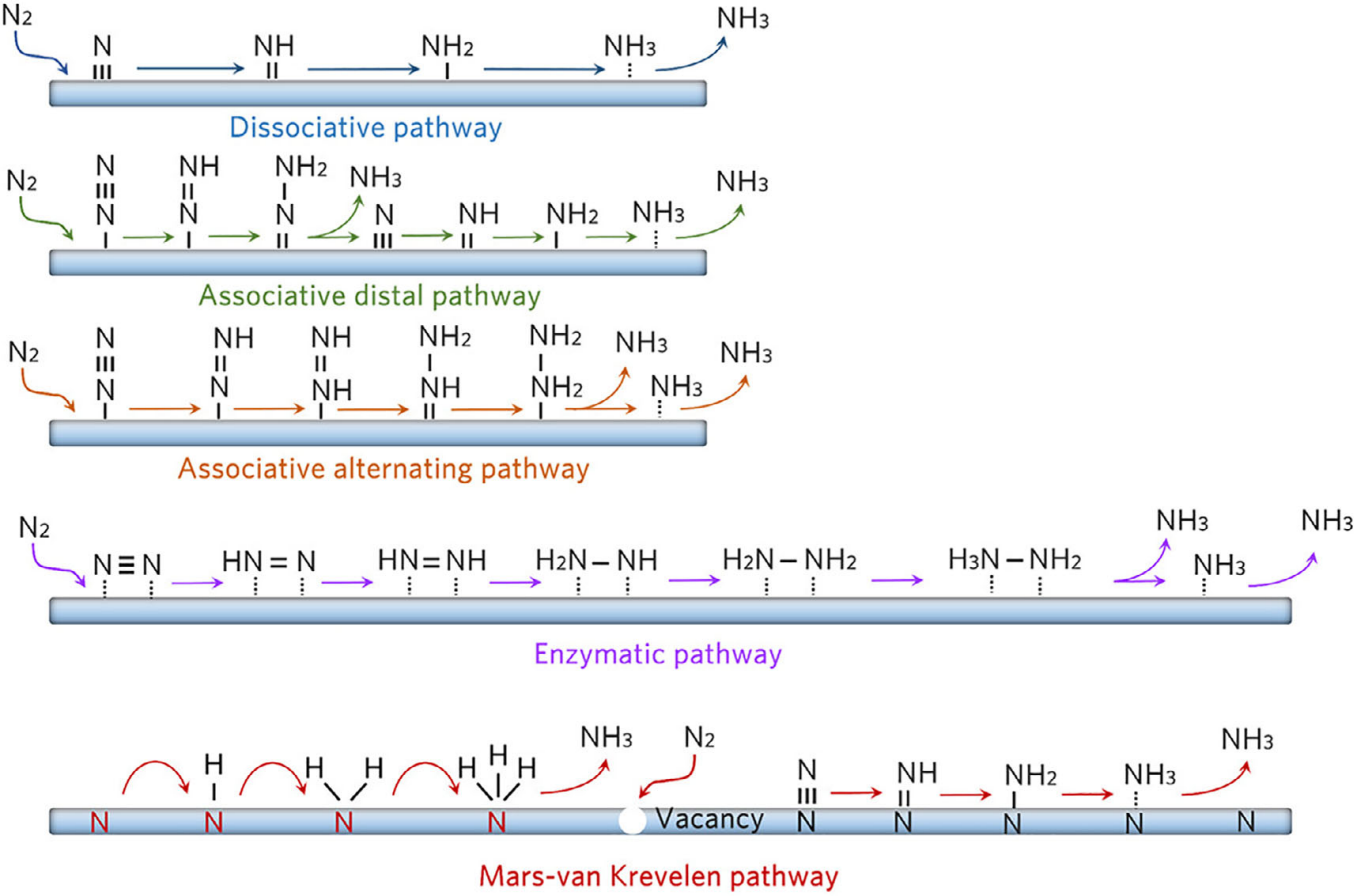
Schematic illustration of the variety of pathways involved in the nitrogen reduction reaction [33].

Similar to other photocatalytic processes, in N_2_ reduction reactions, the electrons in the semiconductor material's VB are excited to the CB by suitable light illumination. The photoinduced electrons in the CB transferred to N_2_ molecules, thereby reducing them to ammonia. At the same time, holes generated in the VB oxidize water molecules. Overall, the nitrogen reduction process for one nitrogen molecule, as represented by the following reactions, involves six electrons, six protons, and six holes ((1), (2), (3)) [30]:

(1) $$N_{2}+6H^{+}+6e^{-}\to 2NH_{3}\;E_{\mathrm{NHE}}^{0}=0.55\;V$$

(2) $$3H_{2}O+6h^{+}\to 6H^{+}+\frac{3}{2}O_{2}\;E_{\mathrm{NHE}}^{0}=1.23\;V$$

(3) $$N_{2}+3H_{2}O\to 2NH_{3}+\frac{3}{2}O_{2}$$

Photocatalytic nitrogen reduction is governed by the relative positions of the photocatalyst's CB and VB with respect to the redox potentials of the participating reactions. For efficient N_2_ photoreduction, the CB potential should be more negative than the thermodynamic potential required for N_2_ reduction, providing sufficient driving force for electron transfer to adsorbed N_2_ molecules, while the VB potential should be sufficiently positive to facilitate water oxidation. However, water oxidation is a kinetically sluggish multielectron process and often suffers from severe charge‐carrier recombination, thereby limiting the overall NRR efficiency. To overcome this limitation, sacrificial reagents (hole scavengers) such as methanol, ethanol, sodium sulfite, and triethanolamine are frequently employed in laboratory‐scale studies. These reagents readily consume photogenerated holes, suppress electron–hole recombination, and increase the availability of electrons for N_2_ reduction, thereby often enhancing NH_3_ production. Nevertheless, the use of sacrificial reagents compromises the practical applicability of the system, as the photocatalytic process is no longer coupled with water oxidation and may complicate accurate assessment of photocatalytic performance. Therefore, although hole scavengers are valuable for investigating the intrinsic activity of photocatalysts and understanding reaction mechanisms, the ultimate objective is to develop efficient photocatalytic systems capable of driving N_2_ reduction under sacrificial‐agent‐free conditions [34, 35]. Furthermore, competing reactions such as the hydrogen evolution reaction (HER), oxygen evolution reaction (OER), and oxygen reduction reaction (ORR) can significantly reduce NRR selectivity and efficiency. Among these, HER is particularly detrimental because it competes directly with N_2_ reduction for photogenerated electrons and is often kinetically favored [36]. Consequently, the development of photocatalysts with efficient charge separation, strong N_2_ adsorption and activation capabilities, and suppressed HER activity is essential for achieving efficient and selective solar‐driven ammonia synthesis.

Theoretical investigations play a crucial role in validating and interpreting experimental observations in bismuth‐based photocatalytic systems by providing atomistic‐level insights into the electronic structure, charge transfer behavior, and reaction mechanisms. Density functional theory (DFT) calculations have consistently demonstrated good agreement with experimental results, particularly in predicting band‐gap energies, defect states, adsorption energies, and catalytically active sites [31, 37]. For instance, theoretical studies on bismuth oxyhalides (BiOX) reveal that oxygen vacancies (OVs) and dopant‐induced intermediate energy states narrow the band gap, enhance visible‐light absorption, and promote charge separation, which correlates well with experimentally observed red‐shifts in absorption spectra and enhanced photocatalytic nitrogen reduction performance. Likewise, calculated adsorption energies and charge–density distributions on vacancy‐rich bismuth surfaces explain the efficient activation of N_2_ molecules and the elongation of the N≡N bond observed experimentally [8].

A representative example is the Fe/Bi_2_O_2.33_ photocatalytic system reported by Zhu et al. [38], where DFT calculations were employed to elucidate the reaction pathway and catalytic mechanism. Theoretical results demonstrated that Fe^3+^ acts as an electron‐accepting center, while Bi^2+^ species facilitate electron donation, thereby promoting charge redistribution and lowering the activation barrier for N_2_ reduction. Furthermore, the calculations revealed significant elongation of the N─N bond upon adsorption and identified the energetically favorable hydrogenation pathway for ammonia formation. Comparative studies with other transition‐metal‐doped Bi_2_O_2.33_ systems (Mn, Co, Ni, Cu, and Zn) showed that Fe/Bi_2_O_2.33_ exhibits the longest activated N─N bond length and the lowest energy barrier for the first hydrogenation step (*NN→*NNH), which is consistent with its superior experimentally observed nitrogen fixation activity. These findings highlight the strong synergy between theoretical predictions and experimental results, demonstrating how computational approaches can rationalize observed photocatalytic performance and guide the design of more efficient bismuth‐based photocatalysts.

## 3 Performance Evaluation of Photocatalytic NRRs

### 3.1 Quantification of Ammonia

As is well known, the photocatalytic reduction of nitrogen molecules by semiconductor materials leads to the formation of ammonia. However, in some cases, the amount of ammonia produced is extremely small. Factors such as low ammonia concentration, foreign impurities, systematic errors, and varying measurement conditions can significantly influence results, often leading to inaccurate performance evaluations. Therefore, a precise and reliable method for determining ammonia concentration is essential to accurately assess the photocatalytic N_2_‐reduction efficiency. In aqueous systems, ammonia typically exists as ammonium ions (${\mathrm{NH}}_{4}^{+}$); therefore, the concentration of ${\mathrm{NH}}_{4}^{+}$ is commonly measured as the main product using different analytical techniques. These measurement methods can be broadly categorised based on their analytical principles, including spectrophotometry, IC, fluorescence analysis, ${\mathrm{NH}}_{4}^{+}$ ion‐selective electrodes, and nuclear magnetic resonance (NMR) spectroscopy.

#### 3.1.1 Spectrophotometry

It is the most widely used method for quantifying ammonia due to its low cost and ease of application. In earlier stages of ammonia measurement by spectrophotometry, Kruse–Mellon [39], salicylate‐hypochlorite [40] and trichloramine methods [41] were used. However, due to their susceptibility to interference from metal cations and their low detection limits, these methods have been replaced by more sensitive, simpler to operate, and more accurate methods, such as Nessler's reagent and indophenol blue methods [42].

Nessler's reagent is one of the most commonly used methods for ammonia detection. It consists of an alkaline solution of potassium tetraiodomercurate (II) (K_2_HgI_4_) and potassium hydroxide (KOH). Under alkaline conditions, mercury and iodide ions react with ammonia to form a reddish–brown complex (Reaction 4), which exhibits a characteristic absorbance peak at approximately 420 nm. The ammonia concentration can thus be quantified by measuring the absorbance at this wavelength. However, the use of toxic mercury, short shelf life (approximately 3–4 weeks), slow color development, and dependence on ambient temperature conditions limit the widespread application of this method for ammonia determination [43].

(4) $$2{\left\{\mathrm{Hg}I_{4}\right\}}^{2-}+{\mathrm{NH}}_{3}+3{\mathrm{OH}}^{-}\to {\mathrm{Hg}}_{2}\mathrm{ON}H_{2}I+7I^{-}+2H_{2}O$$

The indophenol blue method operates on the principle of the Berthelot reaction with detection range from 0 to 0.6 mg NH_3_‐NL^−1^. In which ammonia reacts with phenol and alkaline hypochlorite to form a blue‐colored complex exhibiting an absorption peak around 697 nm (Reaction 5). Sodium nitroprusside acts as a catalyst to enhance the intensity of the color formation, while a citrate buffer is used to maintain the pH stability of the reaction solution. The operational parameters, such as the rate and sequence of phenol and hypochlorite addition, significantly influence the accuracy of the results. Therefore, maintaining a consistent, well‐controlled procedure is essential when employing the indophenol blue method for ammonia determination. Both Nessler's reagent and the indophenol blue methods can be effectively used to determine NH_3_ concentrations in neutral or alkaline solutions, whereas only the Nessler's reagent method is suitable for ammonia detection under acidic conditions. When using Nessler's reagent method in acidic media, special attention should be given to the presence of metal cations, as high concentrations of these ions can interfere with the measurement results; however, such interference is generally negligible in alkaline solutions [44].

(5) $$\begin{array}{ccc} & & {\text{2C}}_{6}H_{5}O^{-}+{\text{NH}}_{3}+{\text{3ClO}}_{3}^{-}+{\text{4H}}_{2}O\\ & & \;\to {\left(C_{12}H_{8}{\text{NO}}_{2}\right)}^{-}+{\text{13OH}}^{-}+{\text{3Cl}}^{-}+{\text{12e}}^{-} \end{array}$$

#### 3.1.2 Ion Chromatography

IC is a form of high‐performance liquid chromatography that separates and detects analytes based on their ionic characteristics. High‐efficiency ion‐exchange chromatography is commonly employed for the detection and quantification of ammonium ions. Typical mobile phases used in IC include tartaric acid, methanesulfonic acid, and dimethylpyridine. Compared to conventional spectrophotometric methods, IC offers several advantages, such as the elimination of toxic and carcinogenic reagents, greater simplicity and convenience, high sensitivity to trace concentrations of ammonium ions (as low as 1–10 µg/L), and compatibility with automated systems. The automation of IC enables efficient and routine analysis of multiple samples at trace levels [45]. However, in some cases, cations with similar retention times, such as Na^+^, K^+^, and Li^+^ can produce overlapping peaks with the ${\mathrm{NH}}_{4}^{+}$ ion, thereby limiting the accuracy of ammonia quantification. This issue can be mitigated by using optimized separation conditions, specialized columns, and advanced detection systems that minimize interference from other ions during NH_4_^+^ determination [42, 46].

#### 3.1.3 Ion‐Selective Electrode Method

Two types of electrodes are commonly used to measure dissolved ammonia: the ammonium ion‐selective electrode (${\mathrm{NH}}_{4}^{+}$‐ISE) and the ammonia gas‐sensing electrode. The ${\mathrm{NH}}_{4}^{+}$‐ISE detects ammonium ions (${\mathrm{NH}}_{4}^{+}$) in solution by measuring the potential difference between the ion‐selective and reference electrode (typically Ag/AgCl), using an ${\mathrm{NH}}_{4}^{+}$‐specific poly(vinyl chloride) (PVC) membrane [47]. The measured potential, governed by the Nernst equation, is proportional to the ${\;\mathrm{NH}}_{4}^{+}$ concentration, with a wide detection range of 0.06–20,000 mg L^−1^ NH_3_‐N, depending on the instrument. However, its performance may be affected by interference from other cations, such as Na^+^ and K^+^ [48, 49]. The ammonia gas‐sensing electrode, on the other hand, measures unionized ammonia (NH_3_) and typically consists of a pH electrode, a hydrophobic gas‐permeable membrane, and a reference electrode [48, 50]. Before measurement, the sample solution is made strongly alkaline (pH > 11) to convert ${\mathrm{NH}}_{4}^{+}$ to NH_3_ (aq), which then diffuses through the membrane into the internal ammonium chloride solution until equilibrium is reached. The resulting pH change in the internal solution is proportional to the NH_3_ concentration [43]. This electrode offers a broad detection range (0.01–17,000 mg L^−1^ NH_3_‐N), though performance decreases at very low ammonia levels. Proper sealing of the measurement system is necessary to prevent NH_3_ loss, but unlike spectrophotometric methods, the gas‐sensing electrode is unaffected by sample turbidity or color, making it suitable for a wide range of environmental and aqueous analyses [46].

#### 3.1.4 NMR Method

Accurate quantification of ammonia in photocatalytic NRRs is often hindered by interference from extraneous nitrogen‐containing species arising from the photocatalyst, nitrogen gas, water source, or surrounding environment, leading to an overestimation of the actual ammonia yield. To address these inaccuracies and verify the genuine source of the produced ammonia, ^15^N isotope‐labelled nitrogen (^15^N_2_) is commonly used in place of ^14^N_2_. Under such conditions, ^1^H NMR spectroscopy serves as an effective analytical technique to identify the origin of ammonia, as the detection of ^15^NH_3_ peaks in the NMR spectra provides conclusive evidence that the ammonia is formed through nitrogen reduction rather than from environmental or catalyst‐derived contaminants [51]. In addition to qualitative confirmation, ^1^H NMR has also been utilized for the quantitative determination of ^15^NH_3_, as demonstrated by Jang et al., who established a calibration curve by correlating the peak areas of standard solutions with their respective concentrations [52].

Before analysis, sample solutions are usually acidified to approximately pH 3 with HCl, converting ^15^NH_3_ into ^15^ ${\mathrm{NH}}_{4}^{+}$ to improve detection sensitivity. The ^1^H NMR technique provides high sensitivity, good reproducibility, and clear discrimination from contaminant ammonia, without the need for the complex chemical treatments often required in spectrophotometric methods [53]. Nonetheless, its use is constrained by the high cost of instrumentation, long acquisition times, and signal interference at ${\mathrm{NH}}_{4}^{+}$ concentrations below 10 µmol L^−1^. In contrast, mass spectrometry offers a more sensitive and accurate approach for detecting and quantifying isotopic ammonia. ^14^NH_3_ and ^15^NH_3_ can be distinguished based on their specific mass‐to‐charge ratios (*m*/*z* 251.0854 and 252.0825, respectively). Due to its excellent sensitivity, selectivity, and quantitative reliability, ultra‐high‐performance liquid chromatography‐mass spectrometry (UHPLC‐MS) is particularly promising for isotope‐tracing experiments and mechanistic investigations in NRRs [30, 53].

#### 3.1.5 Fluorometric Method

This method offers high sensitivity for the detection and quantification of ammonia generated during N_2_ reduction reactions, even at low concentrations (1.0 × 10^−4^–2.8 × 10^−3^ mg L^−1^) [48]. In the fluorometric analysis, the ammonia produced from N_2_ reduction reacts with *o*‐phthaldialdehyde and sulfite to form a highly fluorescent isoindole derivative, as illustrated in Reaction (6). The resulting fluorescent compound exhibits maximum excitation and emission at 362.5 and 423 nm, respectively, enabling the precise determination of ammonia concentration [54, 55]. The presence of amines and amino acids in the solution is typically avoided, as these species can also react with *o*‐phthaldialdehyde and sulfite to yield fluorescent products. To minimize such interference, Zhu et al. developed a modified fluorescence method incorporating a purge‐and‐trap pretreatment prior to measurement and observed that amines and amino acids had negligible effects on ammonia determination using a fluorescence detector [56]. In addition to the above‐discussed ammonia quantification techniques, several other methods, including conductivity, titrimetric, and enzymatic analyses, have also been reported. However, owing to their low sensitivity and relatively complex procedures, these methods are seldom employed in recent studies [42, 46].

(6) $${\text{NH}}_{4}^{+}+C_{6}H_{4}{\text{(CHO)}}_{2}+{\text{SO}}_{3}^{2-}+e^{-}\to C_{8}H_{6}{\text{NSO}}_{3}+{\text{2H}}_{2}O$$

The selection of an ammonia quantification method in NRR studies depends on the expected ${\mathrm{NH}}_{4}^{+}$ concentration and the required sensitivity. The indophenol blue and Nessler's spectrophotometric methods exhibit detection ranges of 0–0.6 and 0.025–5.0 mg L^−1^ NH_3_‐N, respectively, while IC and ${\mathrm{NH}}_{4}^{+}$‐ISE cover broader ranges of 0.02–40 and 0.06–20,000 mg L^−1^ NH_3_‐N, respectively. The NH_3_ gas‐sensing electrode operates over 0.01–17,000 mg L^−1^ NH_3_‐N, whereas fluorometric analysis provides the highest sensitivity (1.0 × 10^−4^–2.8 × 10^−3^ mg L^−1^ NH_3_‐N) and can detect ammonia at nanomolar levels. Although ^1^H NMR does not have a fixed detection range, it offers high sensitivity and enables direct discrimination between ^14^ ${\mathrm{NH}}_{4}^{+}$ and ^15^ ${\mathrm{NH}}_{4}^{+}$, making it particularly valuable for isotope‐labeling experiments. Since ${\mathrm{NH}}_{4}^{+}$ concentrations reported in most NRR studies typically fall within the low µM to several hundred µM range, the indophenol blue method remains the most practical choice for routine quantification, while ^15^N‐assisted ^1^H NMR is recommended for rigorous validation of ammonia production and exclusion of false‐positive results.

### 3.2 Experimental Protocols and Contamination Control in Photocatalytic NRR

Reliable evaluation of photocatalytic NRR performance requires rigorous experimental protocols to minimize false‐positive NH_3_ detection and ensure reproducibility. Particular attention should be given to contamination control, as adventitious NH_3_ and NO_x_ species may originate from laboratory air, glassware, water, feed gases, or catalyst precursors. Therefore, all reaction components should be thoroughly cleaned, ultrapure water should be used, and N_2_ (including ^15^N_2_) and Ar gases should be purified prior to use. Experiments should be conducted in closed reaction systems under controlled temperature conditions, with careful reporting of operational parameters, including catalyst loading, gas flow rate, light intensity, and irradiation time. Appropriate control experiments, including blank, dark, and Ar atmosphere tests, are essential to distinguish genuine photocatalytic N_2_ reduction from background contamination. In addition, photocatalysts synthesized from nitrogen‐containing precursors should be carefully examined for residual nitrogenous species, and their contribution should be assessed through control measurements. To verify the origin of the produced ammonia, isotope‐labeling experiments using purified ^15^N_2_ coupled with analytical techniques such as ^1^H NMR or mass spectrometry are strongly recommended. Furthermore, NH_3_ quantification should be validated using multiple analytical methods whenever possible, and all measurements should be repeated at least three times to ensure statistical reliability and reproducibility [57, 58]. A systematic protocol for the reliable assessment of photocatalytic N_2_‐reduction activity is depicted in Figure 2.

**FIGURE 2:**
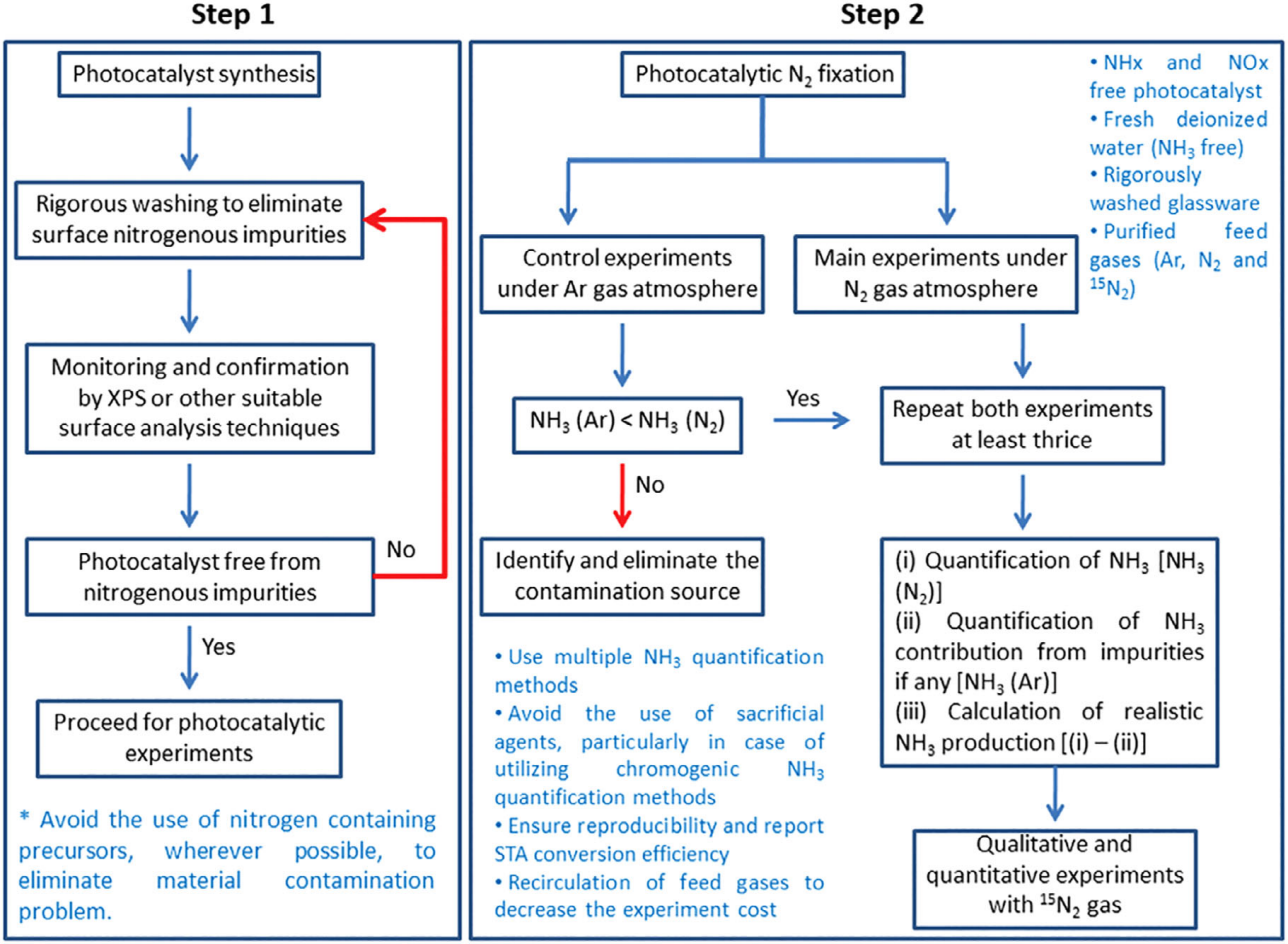
Flowchart summarizing best‐practice guidelines for the accurate evaluation of photocatalytic N_2_ reduction performance. Reprinted with permission from [58]. Copyright 2024, Wiley‐VCH GmbH.

## 4 Photocatalytic Bismuth Chalcogenides for NRR

Photocatalysis has emerged as a highly effective and sustainable approach for the degradation of pollutants. Semiconductor materials play a pivotal role as photocatalysts by facilitating chemical reactions through the absorption of light energy. The photocatalytic efficiency of these materials strongly depends on several intrinsic properties, including morphology, particle size, crystal structure, surface characteristics, and optical behavior [59]. Among various semiconductors, bismuth chalcogenides such as bismuth oxide, bismuth sulfide, and bismuth selenide have attracted significant attention for photocatalytic applications owing to their unique physicochemical properties. These n‐type semiconductors exhibit strong visible‐light responsiveness, a layered structure and possess desirable features such as narrow band gaps, high refractive indices, ion conductivities, dielectric permittivities, and photoconductivities [60]. Such characteristics make them promising candidates for visible‐light‐driven photocatalytic processes, including the degradation of organic pollutants, water splitting, and NRRs.

Bismuth‐based nanostructures have emerged as promising photocatalysts for the NRR to synthesize ammonia under ambient conditions. During N_2_ activation, bismuth as a p‐block element, can participate in π back‐donation interactions that weaken the strong N–N triple bond, thereby facilitating its activation and cleavage [61, 62]. Bi‐based materials also possess a favorable bandgap of approximately 2.3 eV, enabling efficient light absorption. This is attributed to the stereochemically active lone pair of Bi 6s electrons, which interacts with O 2p orbitals, modifying the VB structure, narrowing the bandgap, and enhancing the mobility of photogenerated charge carriers. Theoretical studies further indicate that the empty Bi p orbitals contribute to electronic hybridization near both the conduction band minimum (CBM) and valence band maximum (VBM), supporting effective charge transfer. Bismuth atoms in bismuth‐based photocatalysts localize the density of states near their Fermi level, which can provide more nitrogen molecule active centers by donating p‐electrons [63]. Consequently, bismuth‐based semiconductors demonstrate strong potential for photocatalytic N_2_ reduction [64].

However, pristine bismuth chalcogenides show limited intrinsic activity for nitrogen reduction. Bismuth chalcogenides (Bi_2_O_3_, Bi_2_S_3_, Bi_2_Se_3_, and Bi_2_Te_3_) are generally weak NRR photocatalysts due to several fundamental shortcomings. First, they possess too few surface‐active sites or defects to facilitate effective N_2_ adsorption and activation, a task made difficult by the remarkable stability of the N≡N bond (≈941 kJ/mol) [21]. Second, their CB minima often lie above −0.23 eV versus NHE, the minimum potential required to drive N_2_ reduction to NH_3_, rendering electron transfer thermodynamically unfavorable. For instance, Bi_2_S_3_ has a CBM near −0.1 eV versus NHE, which is insufficient for NRR. Third, pristine bismuth chalcogenides suffer from rapid electron–hole recombination due to limited charge‐separation pathways, reducing the number of electrons available for catalysis. Additionally, their bulk forms typically exhibit low surface areas (< 10 m^2^/g) and expose nonreactive facets, which further restricts their photocatalytic performance [20, 65].

Despite these limitations, the photocatalytic NRR performance of bismuth chalcogenides can be substantially enhanced through rational catalyst‐engineering strategies that facilitate N_2_ adsorption, activation, and reduction. Since the activation of the highly inert N≡N bond represents the primary challenge in photocatalytic nitrogen fixation, various approaches, including defect engineering, heterojunction construction, elemental doping, and integration with porous supports, have been developed to overcome this limitation. In particular, oxygen, sulfur or selenium vacancies generate coordinatively unsaturated Bi sites that serve as favorable adsorption centers for N_2_ molecules. These defect sites facilitate electron transfer from the catalyst surface to the π* antibonding orbitals of adsorbed N_2_, thereby weakening and elongating the N≡N bond and promoting its activation. Likewise, heterojunctions enhance the separation and migration of photogenerated charge carriers, thereby increasing the availability of electrons at active sites for N_2_ reduction. Elemental doping can tailor the electronic structure and surface charge distribution, optimizing the adsorption and conversion of nitrogen‐containing intermediates, while porous and hierarchical architectures provide abundant accessible active sites and enhanced N_2_ diffusion. Collectively, these modifications not only improve light utilization and charge‐carrier dynamics but also lower the kinetic barriers associated with N_2_ activation and subsequent proton‐coupled electron transfer processes, leading to enhanced NH_3_ production under solar irradiation [27, 66, 67]. The influence of these strategies on the photocatalytic NRR performance of bismuth chalcogenides is discussed in detail in the following sections.

### 4.1 Defect‐Engineered Bismuth Chalcogenides

Bi‐based materials readily develop defect structures during synthesis and storage, which profoundly affect their catalytic activity and stability. These defects regulate electron transfer, nitrogen adsorption and activation, light harvesting, and related functionalities. In photocatalysis, defect sites can trap photogenerated electrons, thereby enhancing charge separation. The introduction of defects may also generate discrete electronic states below the CB, facilitating surface charge transfer and improving catalytic efficiency. Bulk defects are generally considered recombination centers for photoexcited electron–hole pairs, restricting charge‐carrier diffusion. By contrast, surface defects often act as adsorption sites, enabling efficient transfer of photogenerated carriers to adsorbed species and accelerating charge separation. Notably, high‐energy surface defects can serve as anchoring sites for the incorporation and stabilization of single atoms [68, 69].

Defects are typically classified by dimensional scale: (i) zero‐dimensional point defects such as vacancies or dopants, (ii) one‐dimensional dislocations including edge and screw types, (iii) two‐dimensional grain boundaries such as tilt, twist, or twin boundaries, and (iv) three‐dimensional bulk defects like lattice distortions or voids [70]. In photocatalytic nitrogen fixation, one‐ and higher‐dimensional defects are seldom emphasized, whereas zero‐dimensional point defects play a dominant role. Accordingly, our focus is restricted to point defects and their influence on NRRs.

#### 4.1.1 Vacancy

Vacancy defects arise when one or more atoms are missing from their lattice sites, resulting in modified electronic structures and altered physicochemical properties. These defects are generally classified as either anion or cation vacancies. In bismuth chalcogenides, anion vacancies correspond to the absence of O, S, or Se atoms, whereas cation vacancies involve the removal of Bi atoms. Extensive research has focused on the role of anion vacancies in catalysts, particularly their influence on catalytic performance [37]. For example, OVs have been shown to facilitate N─N bond cleavage by effectively trapping metastable electrons within the antibonding orbitals of adsorbed nitrogen molecules. Furthermore, it has been demonstrated that the electronic surface properties and bandgap states of photocatalysts can be finely tuned by controlling vacancy concentration [71]. However, too many vacancies hindered charge transfer across the system, thereby reducing the photocatalyst's performance. Therefore, an appropriate number of vacancies is required to enhance the performance. Lan and co‐workers [72] synthesized sulfur‐vacancy‐rich Bi_2_S_3_ nanorods with tunable sulfur vacancy concentrations by varying the solvothermal reaction time. The introduced sulfur vacancies function as active sites for the adsorption and activation of N_2_ molecules. Under full‐spectrum solar irradiation, the synthesized materials achieved an ${\mathrm{NH}}_{4}^{+}$ generation rate of 51.04 µmol g^−1^h^−1^ (Figure 3a). Upon irradiation, Bi_2_S_3_ is photoexcited to generate electron–hole pairs, with electrons migrating to the CB and holes remaining in the VB. The abundant sulfur vacancies in Bi_2_S_3_ act as activation centers, facilitating the adsorption and activation of N_2_ molecules. CB electrons are subsequently transferred to these vacancies, where they reduce N_2_, while VB holes oxidize water molecules to produce H^+^ and O_2_, thereby completing the reduction of N_2_ to NH_3_ (Figure 3b) [73]. This mechanism was confirmed by gas chromatography, which detected only trace amounts of H_2_, while O_2_ concentration increased steadily with reaction time, indicating that H^+^ was used to form ammonia. The nitrogen source in the produced ammonia and the essential role of light were validated through control experiments in which N_2_ was replaced with Ar, and the reaction was performed in the dark with N_2_, both of which yielded negligible activity (Figure 3c). Furthermore, the synthesized Bi_2_S_3_ nanorods exhibited excellent stability, with only a slight decrease in reactivity after four consecutive cycles (Figure 3d).

**FIGURE 3:**
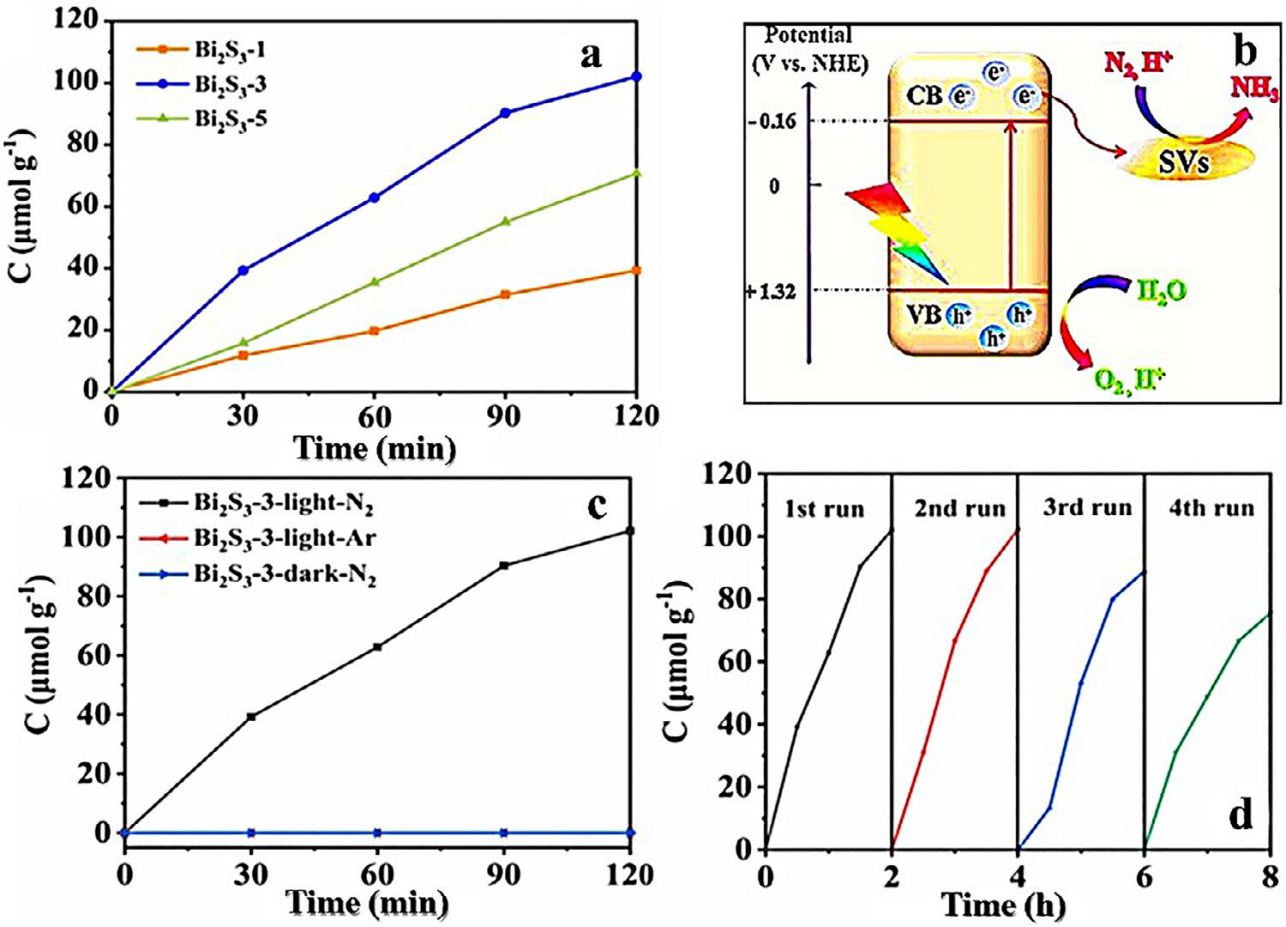
(a) Quantitative determination of the generated NH_3_ under full spectral irradiation. (b) Schematic illustration of the photocatalytic nitrogen fixation mechanism on sulfur‐vacancy‐rich Bi_2_S_3_ nanorods. (c) Control experiments under different conditions. (d) Catalyst cycling tests for Bi_2_S_3_‐3 under full spectral irradiation. Reprinted with permission from [72]. Copyright 2022, Elsevier.

Huang and co‐workers [74] conducted a first‐principles investigation of the role of OVs in δ‐Bi_2_O_3_. Their results demonstrated that a configuration combining <110> and <111> vacancy arrangements is energetically more favorable than structures with isolated <100>, <110>, or <111> vacancies. Analysis of the projected density of states (PDOS) revealed that Bi ions in structures with single <111> vacancy ordering exhibit metallic behavior, whereas mixed 1/3 and 2/3 vacancy distributions lead to semiconducting behavior. The latter configuration produces a band gap of approximately 2.0 eV, suggesting potential applicability of δ‐Bi_2_O_3_ in photocatalytic processes under visible‑light irradiation. The broader implications and underlying mechanisms of vacancy defects are also discussed in the subsequent section of this review.

#### 4.1.2 Doping

Doping is the intentional introduction of guest atoms into a host lattice. It is an effective strategy for tuning the physical and chemical properties of materials, primarily due to differences in electronegativity between the dopant and host atoms. The introduction of dopant atoms can modify surface characteristics and generate additional active sites, thereby enhancing catalytic performance. Typically, doping introduces intermediate energy levels between the CB and the VB, thereby reducing the energy required for electron excitation (Figure 4) [60]. The effectiveness of doping is governed not only by bandgap narrowing but also by improved charge separation and enhanced light absorption. Moreover, doping can promote photocatalytic activity by facilitating more efficient charge‐carrier transfer to the semiconductor surface. Consequently, rational dopant selection combined with strategic morphological control offers a promising route for designing highly efficient photocatalysts for NRRs.

**FIGURE 4:**
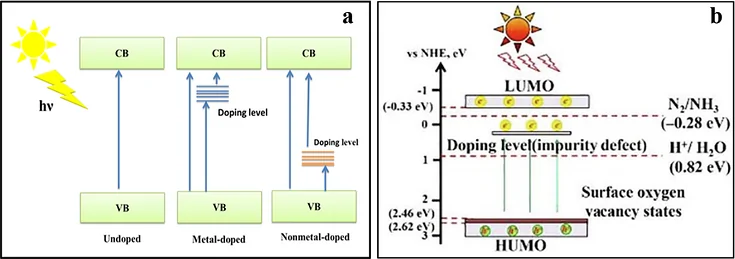
(a) Energy level diagram for metal‐doped and nonmetal‐doped photocatalysts. Reprinted with permission from [60]. Copyright 2024, Elsevier, (b) The proposed photocatalytic nitrogen reduction mechanism by Cu‐doped δ‐Bi_2_O_3_ nanosheets. Reprinted with permission from [75]. Copyright 2022, Elsevier.

Gao and coworkers [75] reported the Cu‐doped δ‐Bi_2_O_3_ nanosheets with OVs that exhibit a high ammonia production rate of 142.8 µmol g^−1^h^−1^ without any sacrificial agent. Cu‐doped δ‐Bi_2_O_3_ followed a mechanism where dopant‐induced electronic modulation and defect engineering jointly enhance nitrogen fixation. Cu incorporation lowered the charge‐transfer resistance and suppressed electron–hole recombination, as evidenced by electrochemical impedance spectroscopy (EIS), photocurrent, and photoluminescence (PL) analyses. The introduction of impurity energy levels and shallow oxygen‐vacancy states narrows the bandgap and shifts the CB to a more negative potential, enabling stepwise electron excitation and efficient charge separation as shown in Figure 4b. Benefiting from the 2D ultrathin morphology, photogenerated electrons rapidly migrated to the surface, where Cu‐related defect sites and OVs serve as active centers for N_2_ adsorption and activation. Because the conduction‐band potential is sufficiently negative for N_2_ reduction, the activated N_2_ molecules are subsequently reduced to NH_3_ by photoexcited electrons, resulting in enhanced photocatalytic nitrogen fixation. In another study, Chu and co‐workers [76] reported enhanced photocatalytic nitrogen reduction performance for Ru‐doped Bi_2_O_3_. Ru‐doped Bi_2_O_3_ containing abundant OVs was synthesized using a rapid Joule heating method. Ruthenium doping increased the density of active sites for nitrogen reduction and suppressed electron–hole recombination, while OVs promoted effective charge separation and facilitated N_2_ adsorption. DFT calculations revealed that the formation of Ru‐O‐Bi bonds and exposed Ru sites in Ru‐Bi_2_O_3_ are energetically favorable for N_2_ adsorption. Furthermore, the alternating nitrogen fixation pathway was identified as the preferred reaction mechanism for nitrogen reduction (Figure 5). The photocatalytic nitrogen reduction rate was 32 µmol g^−1^ h^−1^, which was higher than that of pristine Bi_2_O_3_.

**FIGURE 5:**
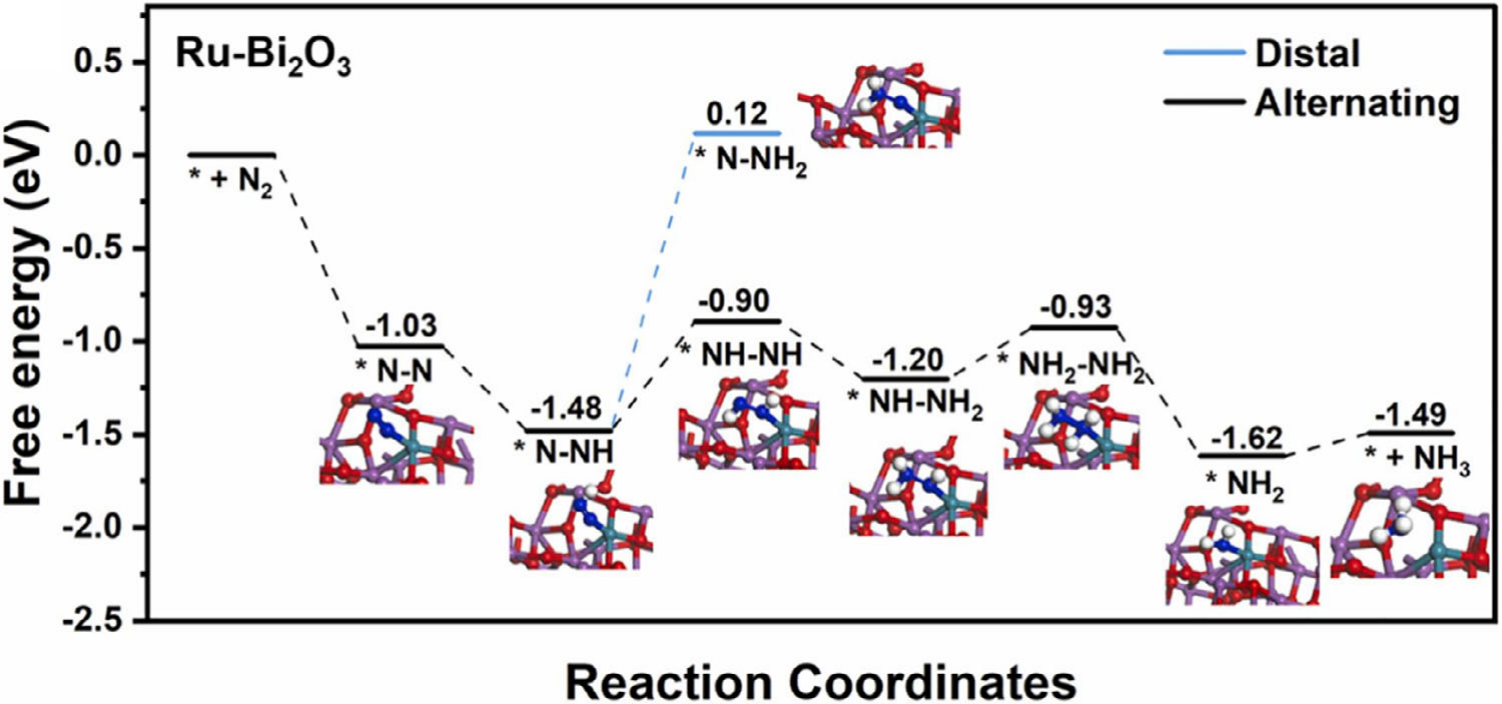
Free energy diagram of NRR on Ru‐Bi_2_O_3_. Reprinted with permission from [76]. Copyright 2025, Elsevier.

Along similar lines, Wu et al. [77] developed a Ti‐doped δ‐Bi_2_O_3_ architecture that exhibits enhanced photocatalytic nitrogen reduction performance. The incorporation of Ti into the Bi_2_O_3_ lattice results in bandgap narrowing, an upward shift of the conduction‐band minimum, and prolonged charge‐carrier lifetime, thereby facilitating efficient light absorption and charge separation. DFT calculations and temperature‐programmed desorption analyses reveal the formation of Ti‐Bi dual‐active sites, which generate a favorable O–N_2_ adsorption configuration and significantly lower the hydrogenation energy barrier of the rate‐determining step (*NN→*NNH) from 2.50 (δ‐Bi_2_O_3_) to 2.13 eV (Ti‐doped δ‐Bi_2_O_3_) (Figure 6a). This improvement is attributed to strong orbital hybridization between the N 2p orbitals and the Bi 6p and Ti 3d orbitals on the Ti‐Bi_2_O_3_ surface. Consequently, end‐on adsorbed N_2_ molecules can undergo both alternating and distal hydrogenation pathways toward NH_3_ formation. First, as shown in Figure 6b, the bridging oxygen of the Ti‐Bi site anchors and activates the N_2_ molecule (Step‐1). Subsequent proton‐electron transfers begin to hydrogenate the adsorbed nitrogen (Steps 2 and 3), forming arrangements such as N_2_H and N_2_H_2_. At this point, the reaction can follow two distinct pathways. In the alternative pathway (Steps 4 and 5), hydrogenation continues on the same nitrogen atom until one NH_3_ molecule is released, after which the remaining nitrogen undergoes further hydrogenation (Steps 9–11) to yield the second NH_3_ molecule. In the distal pathway (Steps 6–8), hydrogenation preferentially occurs on the distal nitrogen atom, leading to its earlier release as NH_3_, while the proximal nitrogen is subsequently reduced in Steps 9 and 10. In both cases, the N─N bond is progressively weakened and eventually cleaved, enabling complete conversion to ammonia. Notably, 10% Ti‐Bi_2_O_3_ delivered an ammonia production rate of 508.6 µmol g^−1^ h^−1^ in the absence of sacrificial agents and maintained a stable activity and structural integrity over at least five consecutive catalytic cycles.

**FIGURE 6:**
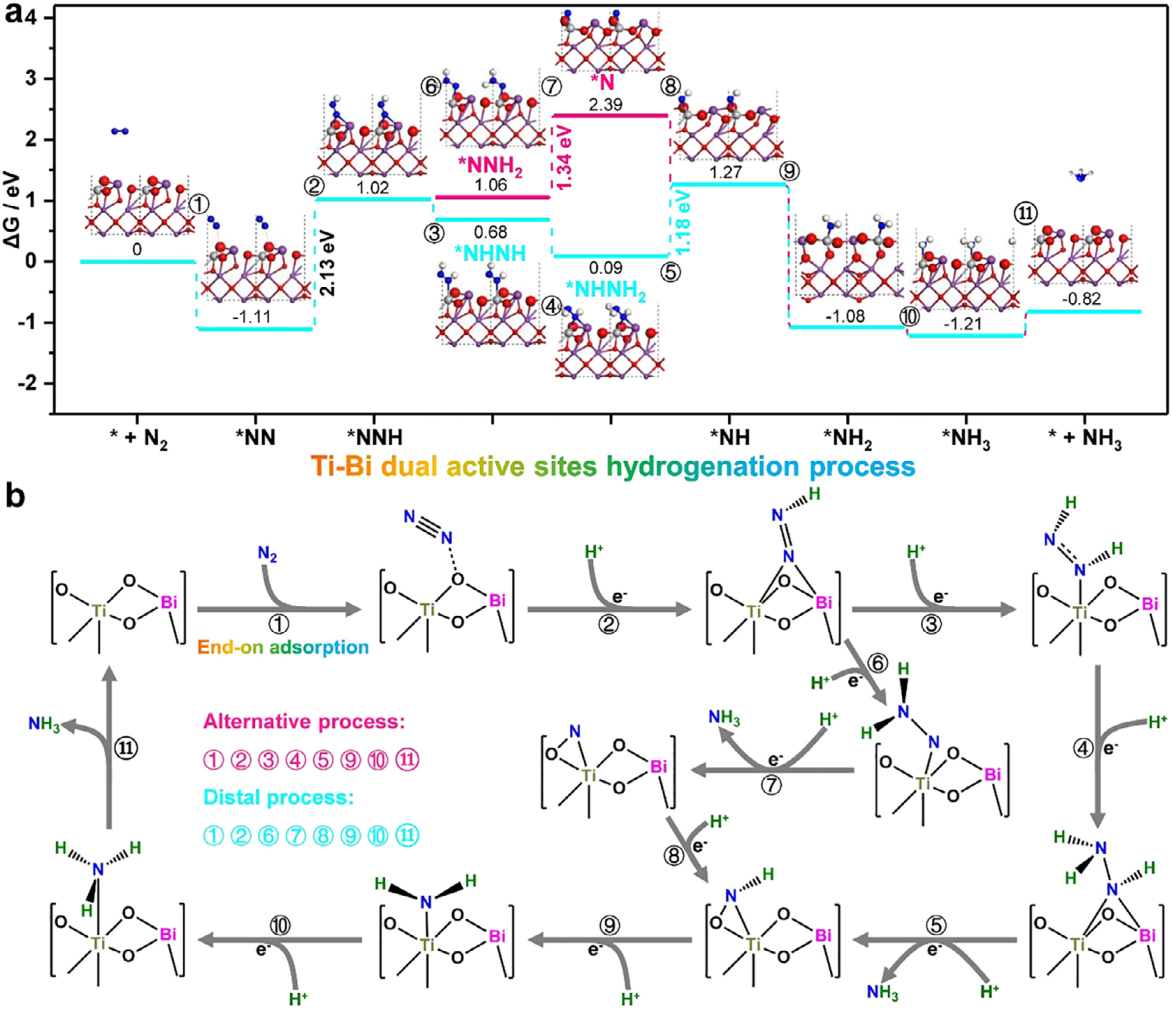
(a) Gibbs free energy profiles of the alternative and distal pathways over Ti‐Bi_2_O_3_. (b) Proposed reaction mechanism diagram. Reprinted with permission from [77]. Copyright 2022, Wiley‐VCH GmbH.

In defect‐engineered bismuth chalcogenides and related bismuth‐based photocatalysts, the combination of heteroatom doping and vacancy engineering has emerged as one of the most effective strategies for enhancing photocatalytic N_2_ reduction. Vacancies serve as active sites for N_2_ adsorption and activation, while dopants can modulate the electronic structure, improve visible‐light absorption, and promote charge‐carrier separation. The synergistic interaction between dopants and defect sites facilitates N_2_ activation and suppresses electron‐hole recombination, thereby enhancing ammonia production and improving photocatalytic stability compared with systems employing either doping or vacancy engineering alone. Ti‐doped δ‐Bi_2_O_3_ with abundant OVs is a representative example demonstrating the effectiveness of this dual‐engineering strategy.

### 4.2 Heterojunctions

Heterojunctions formed by integrating two different semiconductors provide an effective strategy for enhancing photocatalytic activity by facilitating the efficient separation of photogenerated electrons and holes [78]. Most bismuth chalcogenide‐assisted photocatalysts exhibit narrow band gaps, which enable strong absorption in the visible‐light region; however, these narrow band gaps also increase the likelihood of electron–hole recombination, thereby reducing photocatalytic efficiency. Constructing heterojunctions offers an effective solution to this limitation by promoting charge separation and suppressing recombination processes. Bismuth chalcogenide‐based heterojunction systems, which are most effectively used for nitrogen reduction, are categorized into conventional Type‐II, Z‐scheme, and S‐scheme configurations, as illustrated in Figure 7.

**FIGURE 7:**
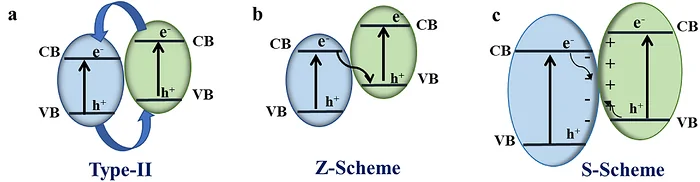
Schematic representation illustrating carrier migrations in conventional (a) Type‐II, (b) Z‐Scheme, and (c) S‐scheme heterojunctions between two semiconductors.

#### 4.2.1 Type‐II Heterojunctions

A type‐II heterojunction configuration is formed when two different semiconductors with unequal Fermi levels come into contact. The difference in Fermi levels, along with variations in work function and the resulting Coulombic interactions, drives charge transfer across the interface until equilibrium is reached [79, 80]. In a type‐II heterojunction, the VB and CB edges of the two semiconductors exhibit a staggered band alignment, as illustrated in Figure 7a. In this arrangement, the CB and VB positions of one semiconductor possess higher reduction and oxidation potentials, respectively, than those of the second semiconductor. Consequently, photogenerated electrons migrate from the semiconductor with a more negative CB to the one with a less negative CB, while photogenerated holes transfer from the semiconductor with a more positive VB to the one with a less positive VB. This spatial separation of electrons and holes across different semiconductors effectively suppresses charge recombination, prolongs carrier lifetimes, and enhances catalytic performance in various applications [60].

From a fundamental perspective, several studies have reported NRRs using bismuth chalcogenide‐based Type‐II heterojunctions. Chen and coworkers [81] synthesized an α‐Bi_2_O_3_/Bi_3_O_4_Br heterojunction using BiOBr and NaOH as the main precursors for photocatalytic nitrogen reduction. The BiOBr nanostructure was stirred with different concentrations of NaOH (0, 0.01, 0.1, 0.2, and 0.5 mol L^−1^) under xenon lamp irradiation for two hours, resulting in α‐Bi_2_O_3_/Bi_3_O_4_Br heterojunctions with varying compositions. The formation of the heterojunction was confirmed by x‐ray Diffraction (XRD), scanning electron microscopy (SEM), high‐resolution transmission electron microscopy (HRTEM), and energy dispersive x‐ray spectroscopy (EDX) analyses, while electron paramagnetic resonance (EPR) and x‐ray photoelectron spectroscopy (XPS) studies verified the presence of surface defects such as lattice oxygen (OL), OVs, and surface hydroxyl groups (OH). The photocatalytic nitrogen fixation performance of the synthesized samples was evaluated in a reaction medium containing 95% water and 5% methanol using Nessler's reagent method and further confirmed by NMR analysis (Figure 8a). Among all samples, BiOBr‐0.1 exhibited the highest ${\mathrm{NH}}_{4}^{+}$ production rate of 238.67 µmol g^−1^ h^−1^, significantly outperforming bare Bi_2_O_3_, Bi_3_O_4_Br, and other heterojunction compositions (Figure 8b). The ${\mathrm{NH}}_{4}^{+}$ generation followed zero‐order kinetics (Figure 8c), indicating that nitrogen adsorption at OV sites governs the reaction. As shown in Figure 8d, the ${\mathrm{NH}}_{4}^{+}$ yield increased with the OV/(OL+OH+OV) ratio, demonstrating a strong positive correlation and highlighting the crucial role of OVs in N_2_ adsorption and activation. The apparent quantum efficiency at 420 nm followed a similar trend, with BiOBr‐0.1 achieving the highest value of 0.049% (Figure 8e). Furthermore, BiOBr‐0.1 retained nearly unchanged activity after four consecutive cycles (Figure 8f), confirming its excellent stability. The superior photocatalytic performance is attributed to the efficient separation of photogenerated charge carriers at the heterojunction interface and the enhanced N_2_ adsorption and activation facilitated by abundant OVs, as shown in Figure 9a.

**FIGURE 8:**
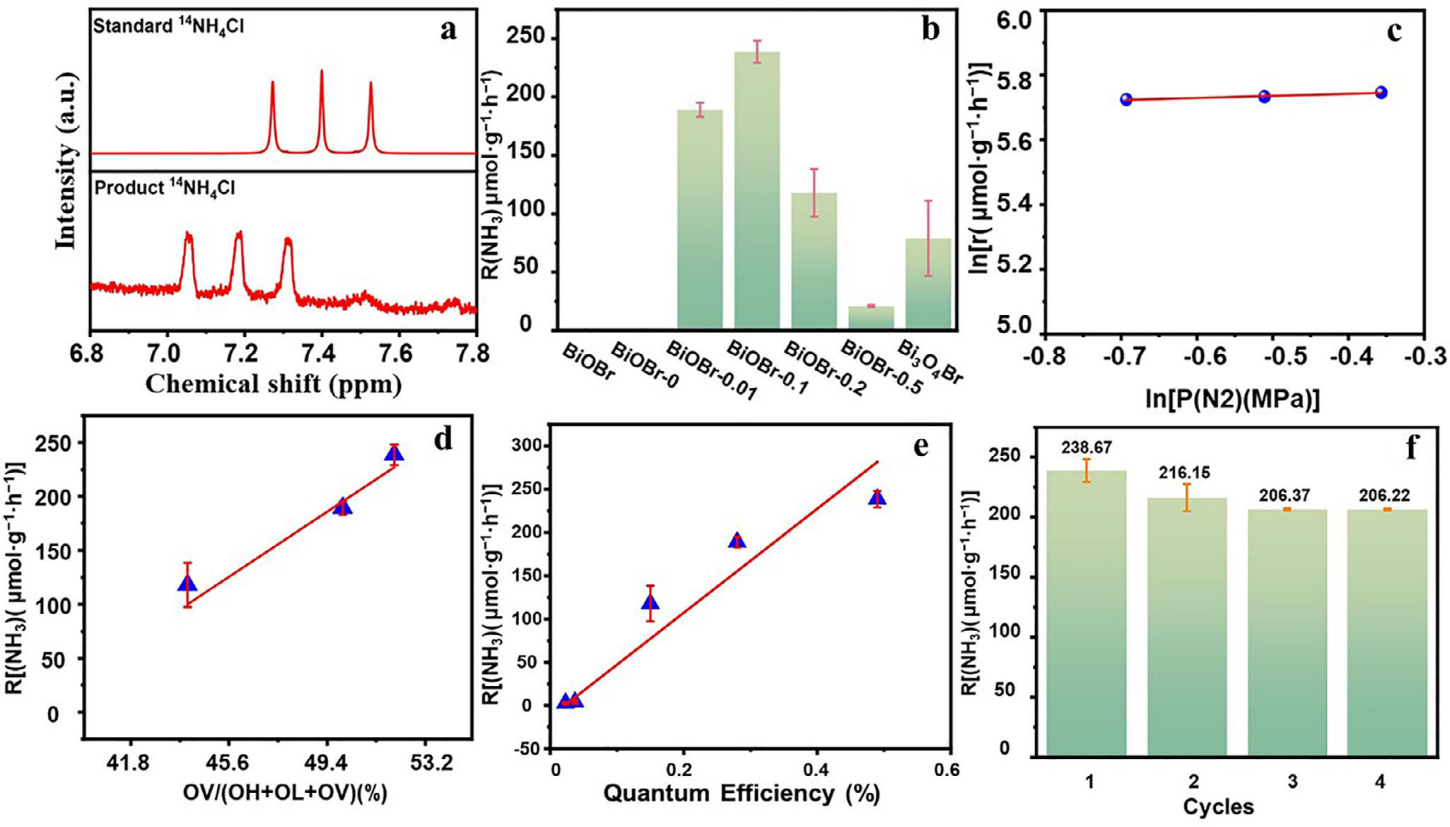
(a) ^1^H NMR spectra of detected ${\mathrm{NH}}_{4}^{+}$ over BiOBr‐0.1 using N_2_ and a methanol (5%, *v*/*v*)/water (95%, *v*/*v*) mixture as feedstock. (b) Photocatalytic ${\mathrm{NH}}_{4}^{+}$ evolution rates of BiOBr, BiOBr‐0, BiOBr‐0.01, BiOBr‐0.1, BiOBr‐0.2, and Bi_3_O_4_Br. (c) ${\mathrm{NH}}_{4}^{+}$ yield over BiOBr‐0.1 as a function of N_2_ pressure (0.5–0.7 MPa). (d) Dependence of ${\mathrm{NH}}_{4}^{+}$ yield on oxygen species ratio (OL, OH, and OV) from XPS. (e) ${\mathrm{NH}}_{4}^{+}$ yield vs. quantum efficiency ratio measured by photoelectrochemical tests. (f) Recyclability of BiOBr‐0.1 for four cycles. Reprinted with permission from [81]. Copyright 2022, Elsevier.

**FIGURE 9:**
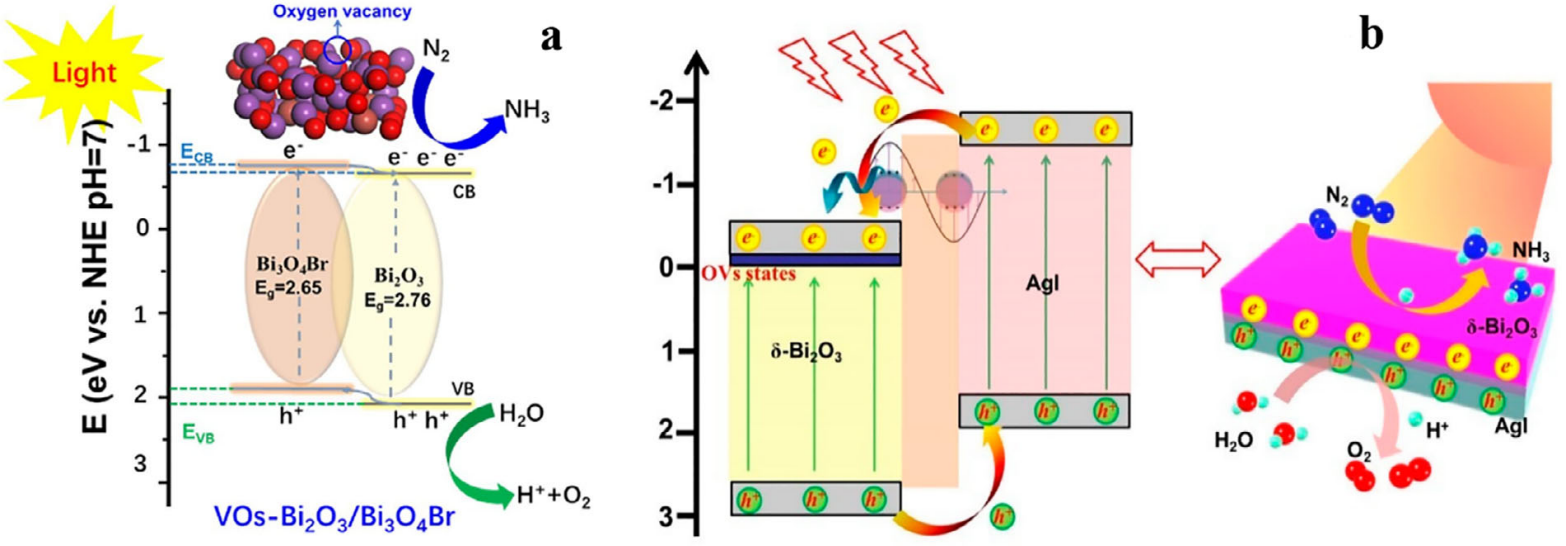
The proposed photocatalytic nitrogen reduction mechanism by (a) α‐Bi_2_O_3_/Bi_3_O_4_Br. (b) Ag/AgI‐δ‐Bi_2_O_3_ photocatalyst [82]. Reprinted with permission from [81]. Copyright 2023, Elsevier.

In another related study, a 2D ultrathin Ag/AgI‐δ‐Bi_2_O_3_ photocatalyst demonstrated a nitrogen fixation performance of 420 µmol L^−1^ g^−1^ h^−1^ in water without the use of any sacrificial agent. The formation of a plasmonic‐sensitized heterojunction, the presence of OVs, and the modified morphology of δ‐Bi_2_O_3_ after the introduction of Ag/AgI nanoparticles collectively enhanced charge separation efficiency, light absorption, and nitrogen adsorption capability, making the system highly effective for photocatalytic ammonia production. Under light irradiation, electrons and holes were produced in the CB and VB of both AgI and Bi_2_O_3_. Due to the staggered band alignment, the photoinduced electrons moved from the CB of AgI to the CB of Bi_2_O_3_, while the holes transferred from the VB of Bi_2_O_3_ to the VB of AgI, as illustrated in Figure 9b. The electrons accumulated in the CB of Bi_2_O_3_ were trapped by OVs and further enriched by hot electrons generated through the photoexcitation of metallic Ag nanoparticles. As a result, the photogenerated charge carriers were efficiently separated. The accumulated electrons subsequently reacted with adsorbed N_2_ molecules and H^+^ to produce ${\mathrm{NH}}_{4}^{+}$, whereas the photogenerated holes oxidised H_2_O to O_2_ [82].

In another study, Lazuli and coworkers [83] reported the synthesis and photocatalytic performance of a Ni‐incorporated ZrO_2_/Bi_2_O_3_ heterojunction. The photocatalytic activity of the Ni‐ZB system under different sacrificial agents, catalysts, controlled experimental conditions, and varying concentrations of triethanolamine (TEOA) is presented in Figure 10. Figure 10a shows the effect of different sacrificial agents in the absence of the photocatalyst. Among the tested agents, TEOA showed the least interference, whereas the others produced significant absorbance signals even in the absence of the catalyst, rendering them unsuitable for photocatalytic N_2_ fixation. Control experiments conducted under an argon atmosphere (Figure 10b) resulted in negligible ammonia production, confirming that N_2_ and water are the primary sources of ammonia. The average ${\mathrm{NH}}_{4}^{+}$ production rate of the Ni‐ZB heterojunction (2379.1 µmol h^−1^g^−1^) was nearly two times higher than that of ZB (1215.5 µmol h^−1^g^−1^), demonstrating the beneficial role of Ni incorporation. Furthermore, the addition of 5% TEOA significantly enhanced the ammonia production rate to 9668.2 µmol h^−1^g^−1^, corresponding to an approximately eight‐fold increase compared to ZB (Figure 10c,d). As shown in Figure 10e,f, the Ni‐ZB heterojunction exhibited the highest activity under light irradiation in the presence of 5% TEOA. This superior photocatalytic performance is attributed to the narrow band gap, the formation of a Type‐II heterojunction between n‐type Bi_2_O_3_ and p‐type Ni‐ZrO_2_, and the abundance of OV sites that facilitate N_2_ adsorption and activation.

**FIGURE 10:**
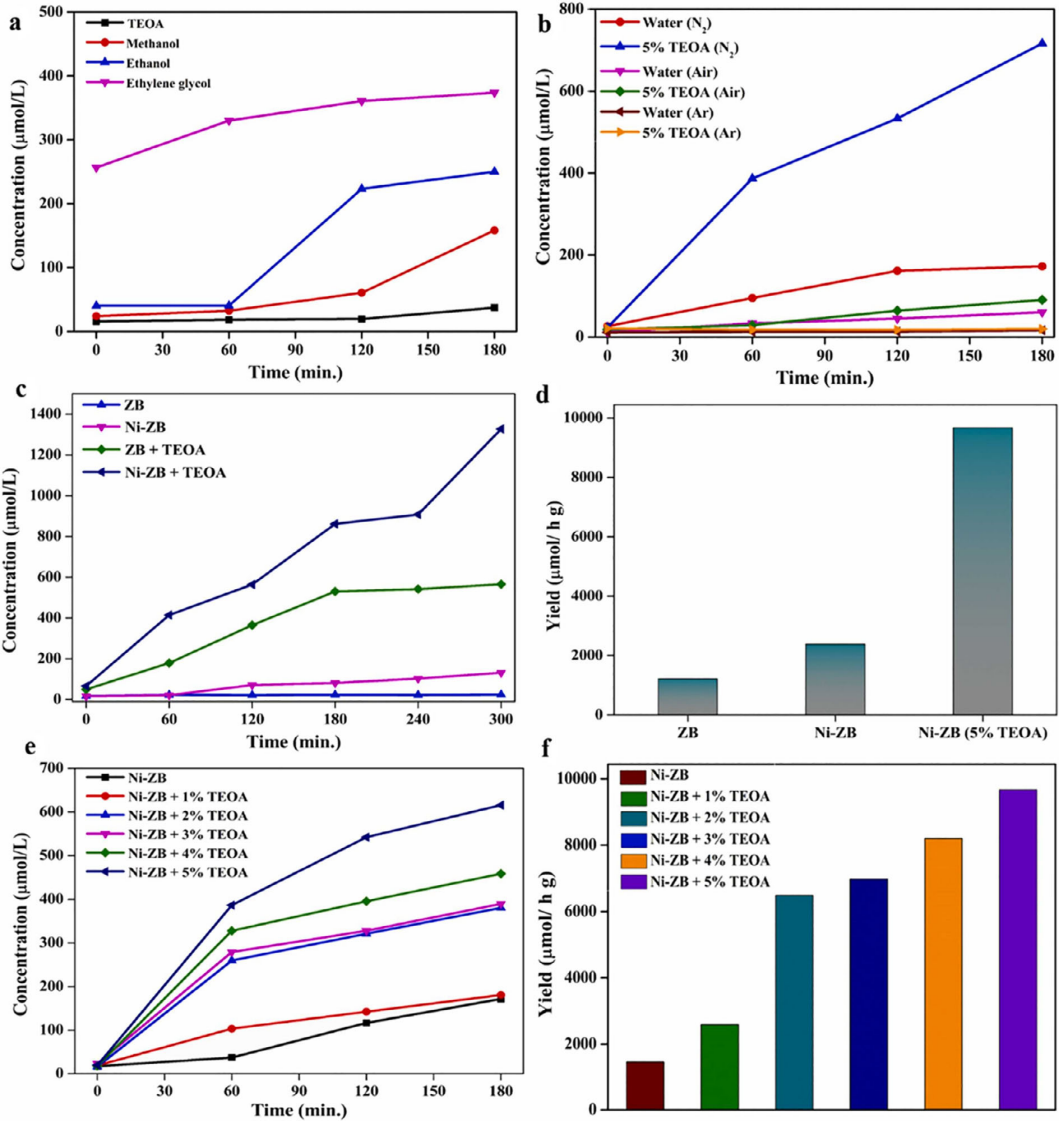
(a) Photoreaction results using different sacrificial agents in the absence of a catalyst; (b) control experiments performed with Ni‐ZB as the catalyst; (c and d) quantitative determination of the generated ammonia in the presence of different catalysts; and (e and f) efficiency obtained with varying concentrations of TEOA, respectively. Reprinted with permission from [83]. Copyright 2023, Elsevier.

#### 4.2.2 Z‐Scheme Heterojunction

In recent years, Z‐scheme heterojunctions have emerged as a highly promising strategy for photocatalytic nitrogen reduction, owing to their ability to mimic natural photosynthesis and achieve efficient charge separation while preserving strong redox potentials. Unlike conventional type‐II heterojunctions, Z‐scheme systems (Figure 7b) facilitate directional electron transfer between semiconductors, ensuring that photogenerated electrons with high reduction potential are retained to drive the activation of inert N_2_ molecules, while holes with strong oxidation potential simultaneously suppress competing side reactions [84]. This synergistic mechanism not only enhances the selectivity and efficiency of ammonia production but also improves stability by reducing charge recombination. Various designs, including direct solid‐state Z‐schemes, mediator‐assisted systems, and facet‐engineered heterojunctions, have demonstrated significant improvements in photocatalytic activity [85]. Shang et al. [86] demonstrated photocatalytic nitrogen reduction using a β‐Bi_2_O_3_/BiOCl composite via a Z‐scheme mechanism. The synthesized heterojunction achieved an ammonia yield of 102.6 µmol/L within 60 min under Xe lamp irradiation, nearly 25 times that of pristine BiOCl. Upon illumination, photogenerated electrons from β‐Bi_2_O_3_ migrated to the VB of BiOCl, where they were subsequently excited to its CB, leaving holes in the VB of β‐Bi_2_O_3_. This process enhanced charge separation efficiency and suppressed recombination. Importantly, the CB potential of BiOCl is more negative than −0.28 eV, which is sufficient to drive the reduction of N_2_ to NH_3_, thereby making the Z‐scheme configuration more favorable than a conventional type‐II heterojunction.

In another study, Zeng et al. [87] developed an oxygen‐vacancy‐rich δ‐Bi_2_O_3_/Bi_2_WO_6_ Z‐scheme heterojunction that achieved a nitrogen fixation yield of 65.6 µmol g^−1^_Cat_, which is about six times higher than that of δ‐Bi_2_O_3_ and 2.8 times higher than that of Bi_2_WO_6_ alone. SEM and TEM analyses of the synthesized material revealed a core‐shell‐type structure, where Bi_2_WO_6_ formed microspheres that were uniformly covered with δ‐Bi_2_O_3_ nanosheets, as shown in Figure 11. The formation of this core‐shell architecture facilitated efficient spatial separation of photogenerated charge carriers (Figure 12a) and provided a directional pathway for charge migration, thereby enhancing electron transport and promoting photocatalytic nitrogen fixation. The combined effects of OVs, a direct Z‐scheme junction, and a core‐shell structure further facilitate charge separation and migration, thereby enhancing the photocatalyst's performance in NRRs.

**FIGURE 11:**
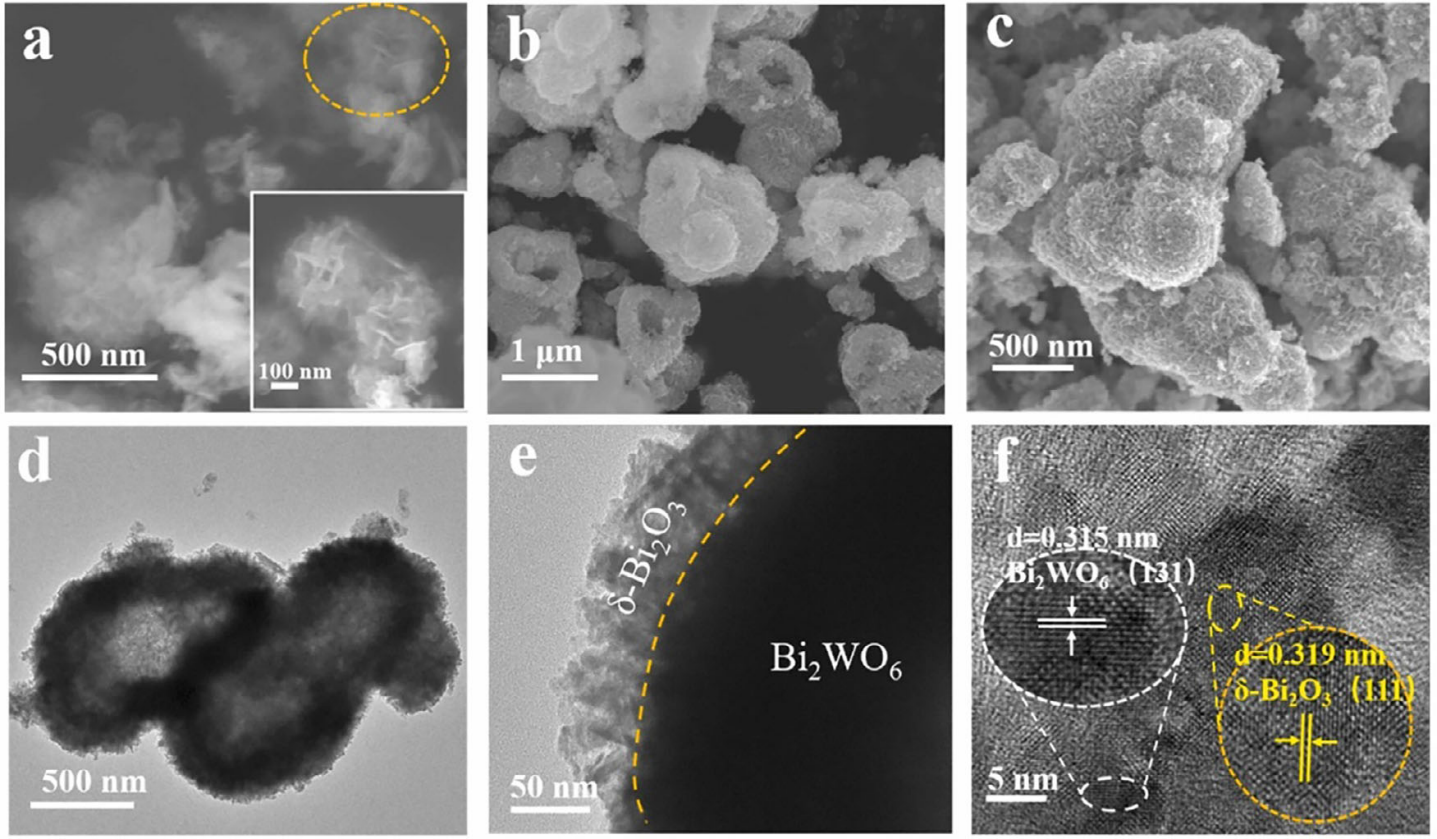
SEM of (a) δ‐Bi_2_O_3_, (b) Bi_2_WO_6_, (c) δ‐Bi_2_O_3_/Bi_2_WO_6_, (d and e) TEM, and (f) HR‐TEM of δ‐Bi_2_O_3_/Bi_2_WO_6_. Reprinted with permission from [87]. Copyright 2024, Elsevier.

**FIGURE 12:**
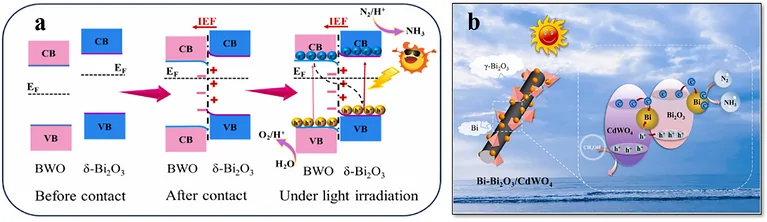
Possible mechanism of (a) δ‐Bi_2_O_3_/Bi_2_WO_6_. (Reprinted with permission from [87]. Copyright 2023, Elsevier.) and (b) Bi‐Bi_2_O_3_/CdWO_4_ Z‐scheme heterojunction for photocatalytic nitrogen fixation reaction. (Reprinted with permission from [88]). Copyright 2023, Elsevier.

Zhao and coworkers [88] reported a mediator‐assisted Z‐scheme heterojunction based on Bi‐Bi_2_O_3_/CdWO_4_ for enhanced N_2_ fixation. The optimized heterostructure exhibited a nitrogen fixation rate of 434.9 µmol L^−1^ g^−1^ h^−1^ under simulated sunlight irradiation, which is 4.9 times higher than that of pure CdWO_4_. In this system, metallic Bi acts as an electron mediator, bridging Bi_2_O_3_ and CdWO_4_, facilitating the recombination of electrons from the CB of CdWO_4_ with holes from the VB of Bi_2_O_3_ under solar illumination due to the internal electric potential gradient. Meanwhile, the electrons and holes with stronger redox potentials are retained in the CB of Bi_2_O_3_ and the VB of CdWO_4_, respectively, where they participate in the photocatalytic nitrogen fixation reaction (Figure 12b). Additionally, the presence of metallic Bi at the Bi_2_O_3_ surface forms a Schottky barrier, which traps electrons from the CB of Bi_2_O_3_ and further enhances charge‐carrier separation efficiency. In another related study, a Bi‐Bi_2_O_3_/KTa_0.5_Nb_0.5_O_3_ (KTN) composite was synthesized via a simple solvothermal method and used for ammonia production, achieving a rate of 466.2 µmol L^−1^ g^−1^ h^−1^. As in previous work, metallic Bi served as a bridge between KTN and Bi_2_O_3_, facilitating rapid charge migration and enhancing charge separation through a Z‐scheme mechanism. Moreover, the presence of metallic Bi suppressed the competing HER, thereby significantly improving the photocatalytic NH_3_ generation [89]. Yang et al. [90] developed a Z‐scheme core‐shell heterojunction based on insoluble‐SiW_11_Fe@δ‐Bi_2_O_3_, which achieved an ammonia production rate of 121 µmol g_cat_^−1^ h^−1^ under simulated sunlight in a nitrogen atmosphere, though the activity decreased in air. In this system, insoluble‐SiW_11_Fe promoted the formation of OVs in δ‐Bi_2_O_3_, thereby enhancing nitrogen chemisorption and facilitating photocatalysis. A detailed investigation using N_2_/O_2_ mixtures revealed that NH_3_/${\mathrm{NH}}_{4}^{+}$ generation strongly depends on the nitrogen volume ratio, a trend also observed in N_2_/Ar mixtures. Notably, in oxygen‐containing atmospheres, H_2_O_2_ was co‐produced alongside NH_3_/${\mathrm{NH}}_{4}^{+}$. The relative product ratio, r(NH_3_/${\mathrm{NH}}_{4}^{+}$)/r(H_2_O_2_), was used to evaluate the reduction capacity of the photocatalyst, indicating that oxygen reduction is more sensitive than nitrogen reduction.

#### 4.2.3 S‐Scheme Heterojunctions

The S‐scheme (Step‐scheme) (Figure 7c) photocatalysis process is a multi‐step mechanism that combines a reduction photocatalyst (RP) and an oxidation photocatalyst (OP), each providing distinct catalytic sites: the RP site activates nitrogen using photogenerated electrons, while the OP site drives proton reduction to hydrogen via photogenerated holes, ultimately leading to ammonia formation [91]. In this configuration, the RP has a higher CB, VB, and Fermi level than the OP. When the two photocatalysts are connected, electrons migrate from the RP to the OP due to differences in Fermi levels and work functions, making the RP positively charged and the OP negatively charged. This charge redistribution induces downward band bending in the OP and upward band bending in the RP, generating an internal electric field that selectively recombines low‐energy carriers while preserving high‐energy electrons and holes for strong redox reactions when irradiated with light of respective energy [92, 93]. Unlike type‐II heterojunctions, which achieve charge separation but weaken the redox potential due to carrier relaxation into lower‐energy states, the S‐scheme maintains both efficient charge separation and strong redox activity [94]. Compared with the classical Z‐scheme, which requires precise band alignment and direct recombination of electrons from one semiconductor with holes to another, the S‐scheme achieves a similar level of strong redox capability more flexibly through built‐in electric fields and band bending, making it a more efficient and versatile design [95].

Zhang et al. [96] reported an S‐scheme Bi_2_S_3_/OV‐Bi_2_MoO_6_ heterostructure that enabled efficient photocatalytic ammonia production, achieving a rate of 126 µmol g^−1^_cat_ under visible light irradiation for 2.5 h. In this system, the VB and CB of Bi_2_S_3_ exhibit higher Fermi levels and lower work functions than those of Bi_2_MoO_6_. When the two semiconductors come into contact, electrons spontaneously transfer from Bi_2_S_3_ to Bi_2_MoO_6_ until their Fermi levels equilibrate. As a result, Bi_2_S_3_ becomes positively charged while Bi_2_MoO_6_ acquires a negative charge. This charge redistribution causes upward band bending in Bi_2_S_3_ and downward band bending in Bi_2_MoO_6_, thereby generating an internal electric field across the interface. Upon visible‐light irradiation, both semiconductors are photoexcited to produce electron‐hole pairs. The internal electric field accelerates the recombination of electrons in Bi_2_MoO_6_ with holes in Bi_2_S_3_, facilitating direct S‐scheme charge transfer. Consequently, the photogenerated electrons in the CB of Bi_2_S_3_ selectively reduce N_2_ to NH_3_/ ${\mathrm{NH}}_{4}^{+}$, while the holes in the VB of Bi_2_MoO_6_ oxidize H_2_O to O_2_, as illustrated in Figure 13. In situ ESR measurements further confirmed the involvement of ROS, specifically superoxide radicals ($.O_{2}^{-}$ and hydroxyl radicals (**^·^** OH). From these results, the VB and CB associated oxidation and reduction half‐reactions for the NRR are outlined below:

(7) $$3H_{2}O\to \frac{3}{2}O_{2}+6H^{+}+6e^{-}\left(\text{Oxygen evolution reaction}\right)$$

(8) $$N_{2}+6H^{+}+6e^{-}\to 2NH_{3}\left(\text{Nitrogen reduction reaction}\right)$$

(9) $$N_{2}+3H_{2}O\to 2NH_{3}+3/2O_{2}\left(\text{Overall reaction}\right)$$

**FIGURE 13:**
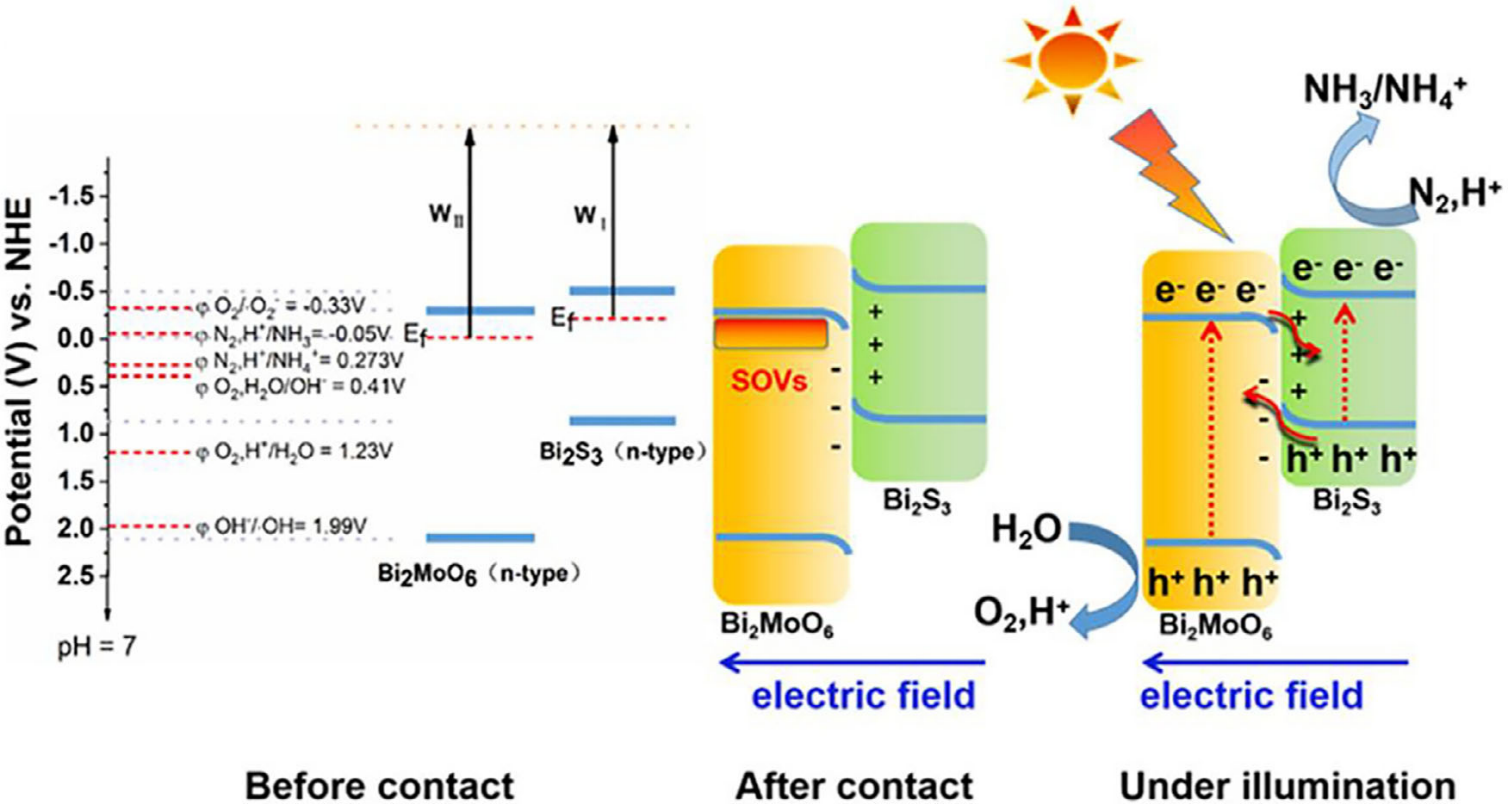
Schematic illustration of the band energy alignment and the separation and migration of photogenerated electron–hole pairs in the Bi_2_S_3_/Bi_2_MoO_6_ hybrid under visible‐light irradiation. Reprinted with permission from [96]. Copyright 2022, Elsevier.

Similarly, Yuan and colleagues [97] developed an S‐scheme Bi_2_O_3_/CdMoO_4_ heterojunction that significantly enhanced photocatalytic nitrogen fixation, achieving a rate of 323 µmol L^−1^ g^−1^ h^−1^, along with efficient tetracycline degradation. The study revealed that dispersing Bi_2_O_3_ nanoparticles across the polymeric CdMoO_4_ surface improved light absorption and facilitated the formation of a tightly bound S‐scheme heterojunction. This heterojunction generated a built‐in electric field that promoted charge separation, thereby increasing the efficiency of electron–hole pair migration and boosting the overall photocatalytic performance of the Bi_2_O_3_/CdMoO_4_ composite in nitrogen fixation reactions. As shown in Figure 14a,b, the photocatalytic activity of the individual components and the Bi_2_O_3_/CdMoO_4_ heterojunction under different conditions demonstrated that the heterojunction with 2% Bi_2_O_3_ composition exhibited the highest activity under a nitrogen atmosphere and 5% methanol as a sacrificial agent (Figure 14d), along with excellent photostability (Figure 14c). In a recent study, a hierarchical 1D/2D Bi_2_O_3_/Bi_4_O_5_I_2_ S‐scheme heterojunction was synthesized via a precipitation‐pyrolysis method. The prepared heterostructure achieved an ammonia production rate of 33.62 µmol g^−1^ h^−1^, which is 2.93 and 2.08 times higher than that of pristine Bi_2_O_3_ and Bi_4_O_5_I_2_, respectively. The superior photocatalytic performance was attributed to enhanced charge separation and accelerated charge transfer driven by the internal electric field established within the S‐scheme heterojunction. This mechanism is strongly supported by in situ XPS analysis, radical trapping experiments, and DFT simulations [98].

**FIGURE 14:**
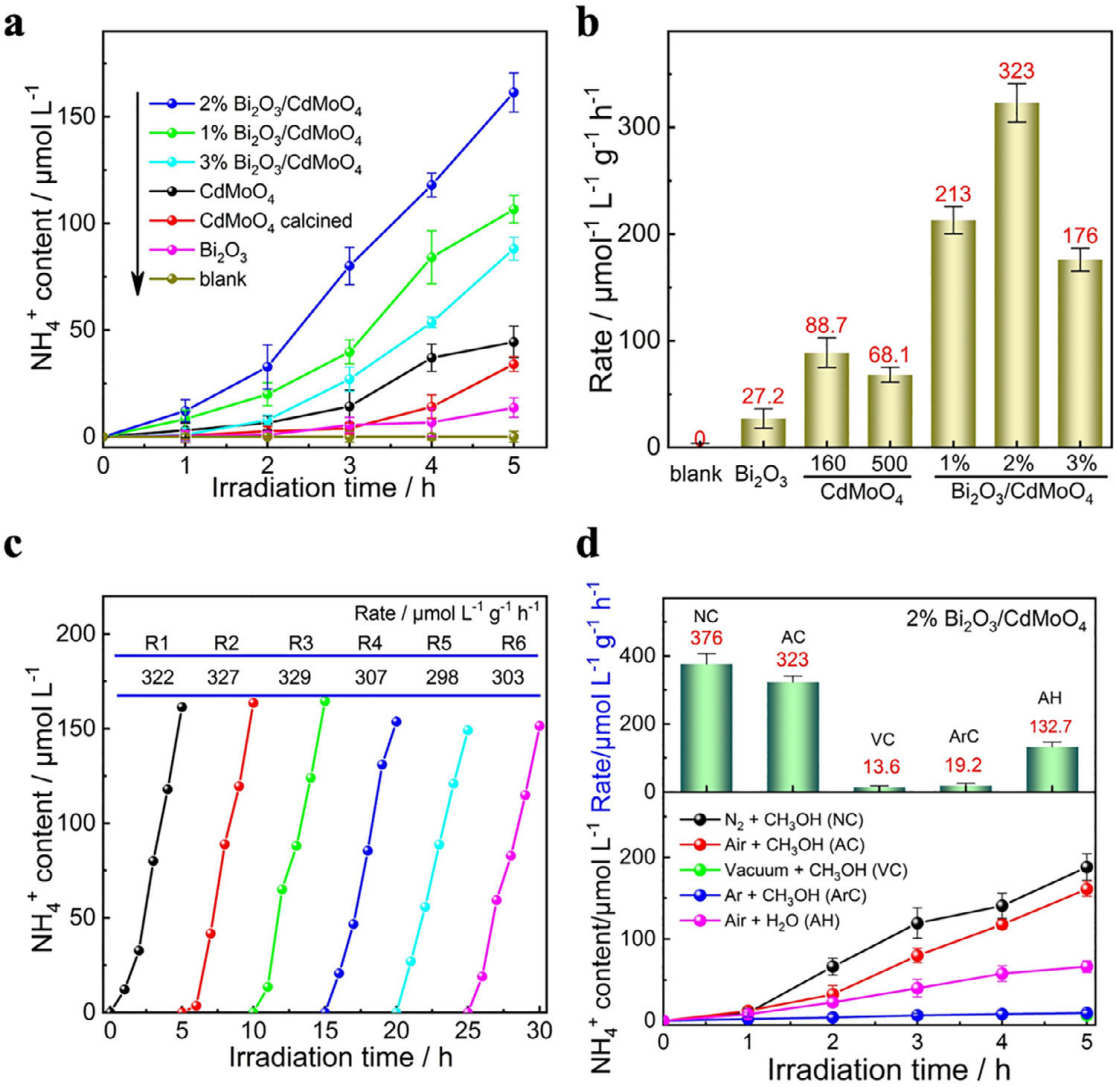
Photocatalytic performance of CdMoO_4_ and Bi_2_O_3_/CdMoO_4_ composites under simulated solar irradiation (a), the corresponding NH_3_ generation rate (b), the cycling stability of 2% Bi_2_O_3_/CdMoO_4_ (c), and its photocatalytic behavior under different atmospheric conditions (d). Reprinted with permission from [97]. Copyright 2023, Elsevier.

According to the reviewed literature, Z‐scheme heterojunctions represent one of the most promising strategies for photocatalytic nitrogen reduction. Unlike conventional Type‐II heterojunctions, which promote charge separation at the expense of redox potential, Z‐scheme systems retain highly reducing electrons and strongly oxidizing holes while facilitating efficient charge‐carrier separation. This enables the effective activation of the chemically inert N_2_ molecule and suppresses competing side reactions, thereby enhancing photocatalytic activity and selectivity. Similarly, S‐scheme heterojunctions retain strong redox properties through internal electric fields and band bending. However, Z‐scheme systems have been more studied and attracted particular attention due to their ability to simultaneously achieve efficient charge separation and strong redox power, making them highly effective candidates for photocatalytic NRR applications.

### 4.3 Bismuth Chalcogenides Embedded in Porous or Hybrid Hosts

Metal–organic frameworks (MOFs) and covalent organic frameworks (COFs) are increasingly recognised as promising photocatalytic materials. MOFs, a class of coordination polymers, exhibit distinctive properties such as high specific surface area, tunable pore structures, and abundant active sites [99]. However, during photocatalytic processes, photogenerated charge carriers migrate through these active centers, where recombination often occurs. This recombination, together with poor water stability and limited recyclability, restricts their photocatalytic applications. To address these challenges, researchers have introduced covalent linkages between MOFs and COFs by incorporating additional substances that act as charge‐migration channels, thereby reducing recombination and enhancing performance [100]. The construction of heterojunctions between MOFs and semiconductors is a highly effective strategy for enhancing photocatalytic nitrogen fixation [101].

Meng and colleagues [102] developed a ZnCdS@CAU‐17 heterojunction incorporating Bi_2_S_3_ as an intermediate layer. CAU‐17, a Bi‐based MOF, features Bi^3+^ ions coordinated with nine oxygen atoms, forming a complex three‐dimensional open framework. This framework contains two hexagonal, two rectangular, and one triangular channel, which facilitate nitrogen adsorption and act as carrier pathways. Upon sunlight irradiation, photogenerated electrons in the CB of CAU‐17 are transferred to the VB of Bi_2_S_3_. Subsequently, electrons migrate from the CB of Bi_2_S_3_ to the VB of ZnCdS, resulting in efficient charge separation. The accumulated electrons in the CB of ZnCdS drive the reduction of N_2_ to ammonia, while holes in the VB of CAU‐17 react with OH^−^ to produce O_2_ and H_2_O. The photocatalytic nitrogen fixation rate of ZnCdS@CAU‐17 heterojunction was 46.96 µmol g^−1^ h^−1^ under simulated sunlight, which was 6.8 and 6.2 times that of ZnCdS and CAU‐17, respectively. In another study, Chen and colleagues [103] developed a highly dispersed Bi_2_S_3_ system supported on a porous porphyrin‐based MOF using a pre‐coordination and in situ sulfurization strategy (Figure 15a). The structure and morphology of the Bi_2_S_3_@PCN‐2 photocatalyst, shown in Figure 15b, reveal the uniform dispersion of Bi_2_S_3_ nanorods (50–60 nm) on cubic PCN crystals (500–800 nm), forming a stable heterojunction while preserving the PCN framework. In contrast, insufficient loading resulted in wrinkled surfaces, whereas excessive loading caused partial framework collapse and the formation of enlarged nanorods. TEM and HRTEM analyses confirmed the close interfacial coupling and clear lattice fringes with a spacing of 0.36 nm, corresponding to the Bi_2_S_3_ (130) plane, while elemental mapping demonstrated the homogeneous distribution of N, S, O, Bi, and Zr throughout the structure. The optimized catalyst exhibited an ammonia production rate of 3880 µg g^−1^ h^−1^ and achieved 96.4% degradation of bisphenol A. Performance enhancement resulted from the synergy between the porphyrin rings and the uniformly dispersed Bi_2_S_3_ nanorods, which broadened visible‐light absorption and facilitated efficient generation and separation of photogenerated electron‐hole pairs, thereby improving both nitrogen fixation and pollutant degradation activities.

**FIGURE 15:**
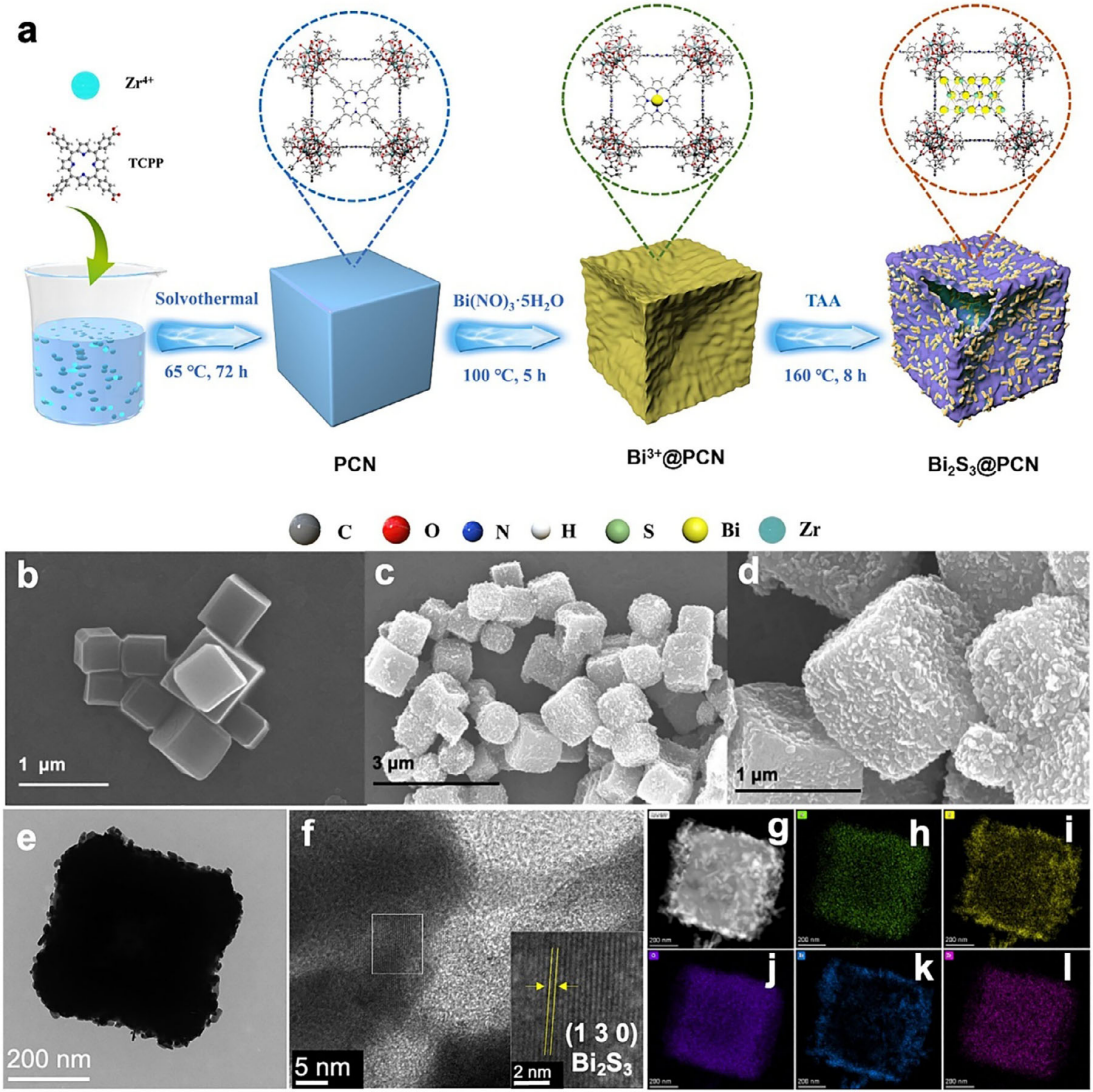
(a) Schematic illustration of the synthesis of the Bi_2_S_3_@PCN‐2 photocatalyst; SEM images of (b) PCN and (c and d) Bi_2_S_3_@PCN‐2; (e) TEM and (f) HRTEM images of Bi_2_S_3_@PCN‐2; (g) HAADF‐STEM image and the corresponding (h–l) elemental mapping images. Reprinted with permission from [103]. Copyright 2024, Elsevier.

Building on these advances, a carbon‐supported Bi_2_O_3_‐BiOCl‐Bi heterostructure was synthesized via a simple hydrothermal method. This system delivered a nitrogen reduction yield of 400 µg L^−1^ under visible‐light irradiation, which was 3.6 times higher than that of pure BiOCl. The enhanced activity was attributed to carbon channels that facilitated electron transport between Bi_2_O_3_ and BiOCl, as well as surface OVs that promoted light absorption and surface reactions. Moreover, the formation of a Schottky junction between metallic Bi and BiOCl further improved charge separation, thereby boosting nitrogen fixation efficiency (Figure 16) [104]. A ternary photocatalyst was developed by loading Bi_2_O_2_CO_3_ and Bi_2_O_3_ nanoparticles onto g‐C_3_N_4_ nanosheets. Among them, the ternary heterostructure showed the highest ammonia production under simulated solar light, with activity enhancements of 7.98, 2.19, and 4.43‐fold compared to pristine g‐C_3_N_4_, g‐C_3_N_4_ nanosheet, and Bi_2_O_2_CO_3_/Bi_2_O_3_, respectively. PL and EIS analyses confirmed improved charge separation and transfer. The superior performance was attributed to the formation of a tandem p‐n‐n heterojunction between p‐type Bi_2_O_3_ and n‐type g‐C_3_N_4_ nanosheets and Bi_2_O_2_CO_3_, which facilitated efficient electron migration for nitrogen photoreduction [105].

**FIGURE 16:**
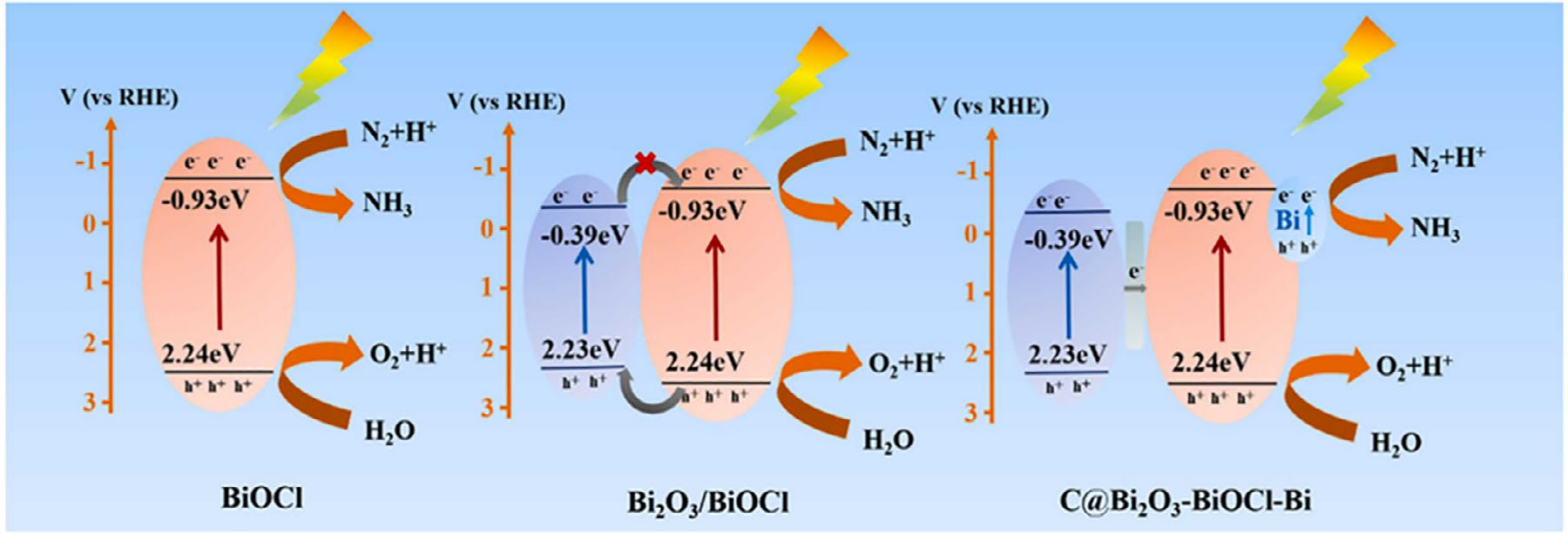
Schematic illustration of the photocatalytic mechanism and charge transfer pathways in the heterojunction systems of BiOCl, Bi_2_O_3_/BiOCl, and C@Bi_2_O_3_‐BiOCl‐Bi. Reprinted with permission from [104]. Copyright 2024, Elsevier.

The integration of bismuth chalcogenides with porous and hybrid hosts demonstrates that embedding these materials within frameworks such as MOFs, carbon‐based matrices, and layered hybrid structures can significantly enhance their photocatalytic nitrogen reduction performance. These hosts provide a high surface area, improved dispersion of active sites, and efficient charge transport pathways, while also enhancing structural stability. Furthermore, the porous architecture promotes N_2_ adsorption and activation, whereas the hybrid framework suppresses nanoparticle agglomeration and improves long‐term durability. Consequently, incorporating bismuth chalcogenides into porous or hybrid hosts is an effective strategy to simultaneously enhance photocatalytic activity, charge separation, and operational stability.

To facilitate a direct comparison of the effectiveness of different photocatalyst engineering strategies, the benchmark NRR performances reported in the literature are summarized in Table 1. Among vacancy‐engineered systems, sulfur‐vacancy‐rich Bi_2_S_3_ nanorods exhibited an NH_3_ production rate of 51.04 µmol g^−1^h^−1^, highlighting the role of surface defects in promoting N_2_ adsorption and activation. In the elemental doping category, Ti‐doped δ‐Bi_2_O_3_ achieved a significantly higher NH_3_ production rate of 508.6 µmol g^−1^h^−1^ without the use of sacrificial reagents, demonstrating the effectiveness of electronic structure modulation. For heterojunction‐based photocatalysts, Ag/AgI‐δ‐Bi_2_O_3_ represented one of the best‐performing type‐II systems with an NH_3_ yield of 420 µmol g^−1^h^−1^, whereas Ni‐ZrO_2_/Bi_2_O_3_ exhibited an exceptionally high activity of 9668.2 µmol g^−1^h^−1^ in the presence of 5% TEOA. Among Z‐scheme photocatalysts, Bi‐Bi_2_O_3_/CdWO_4_ and Bi‐Bi_2_O_3_/KTa_0.5_Nb_0.5_O_3_ achieved NH_3_ production rates of 434.9 and 466.2 µmol g^−1^h^−1^, respectively, while the S‐scheme Bi_2_O_3_/CdMoO_4_ system delivered 323 µmol g^−1^h^−1^. Hybrid host‐supported materials also demonstrated remarkable performance, with Bi_2_S_3_@PCN achieving an NH_3_ production rate of 3880 µmol g^−1^h^−1^. Overall, heterojunction‐based photocatalysts generally exhibit superior NRR performance owing to their enhanced charge separation and utilization of photogenerated carriers, whereas vacancy engineering primarily improves N_2_ adsorption and activation, and elemental doping effectively tailors the electronic structure and charge‐transport properties. The benchmark performances summarized in Table 1 further suggest that integrating multiple modification strategies, such as defect engineering, doping, and heterojunction construction, often leads to the highest photocatalytic NRR activities, highlighting these approaches as promising directions for future catalyst design.

**TABLE 1:** Bismuth chalcogenides‐based photocatalysts for nitrogen reduction.

Sr. no	Catalysts	Strategy used	Ammonia production rate (µmol g^−1^ h^−1^)	Light source and sacrificial agent	Measurement method	Reference
1.	Ru‐Bi_2_S_3_	Doping + vacancies	32	300 W Xe lamp	Nessler's reagent	[76]
2.	α‐Bi_2_O_3_/Bi_3_O_4_Br	Type‐II	238.67	300 W Xe lamp	Nessler's reagent	[81]
3.	Ag/AgI‐δ‐Bi_2_O_3_	Type‐II + oxygen vacancies	420	400 W Xe lamp	Metrohm 833 ions chromatography	[82]
4.	Bi‐Bi_2_O_3_/CdWO_4_	Z‐scheme	434.9	300 W Xe lamp, 5% methanol	Nessler's reagent	[88]
5.	Bi‐Bi_2_O_3_/KTa_0.5_Nb_0.5_O_3_	Z‐scheme	466.2	300 W Xe lamp, methanol	Nessler's reagent	[89]
6.	Bi_2_O_3_/Bi_4_O_5_I_2_	S‐scheme	33.62	300 W Xe lamp	Nessler's reagent	[98]
7.	ZnCdS‐Bi_2_S_3_@CAU‐17	Embedded in the host	46.96	Xe lamp	Ions chromatography	[102]
8.	BrO_3_^−^‐modified Bi_2_O_3_/Bi(OH)_3_	Type‐II	45.28	Xe Lamp (Visible)	Indophenol blue method	[106]
9.	Ultrathin Ag‐δ‐Bi_2_O_3_ sheets	Vacancy	1.7	400 W Xe lamp, light below 400 nm	Nessler's reagent spectrophotometry	[107]
10.	g‐C_3_N_4_ nanosheets/Bi_2_O_2_CO_3_/Bi_2_O_3_	Tandem p‐n‐n heterojunction	4064	500 W Xe lamp	Nessler's reagent	[105]
11.	β‐Bi_2_O_3_/BiOCl	Z‐scheme	102.6	300 W Xenon lamp	Nessler's reagent	[86]
12.	δ‐Bi_2_O_3_/Bi_2_WO_6_	Z‐Scheme	37.8	300 W Xenon lamp	Nessler reagent	[87]
13.	β‐Bi_2_O_3_/BiOCOOH	Oxygen vacancies + heterojunction	65.56	300 W Xenon lamp, methanol	Nessler's reagent	[108]
14.	Cu‐δ‐Bi_2_O_3_	Oxygen vacancies + doping	142.8	300 W Xenon lamp	Metrohm 833 ions Chromatography	[75]
15.	Ti‐Bi_2_O_3_	Doping	508.6	300 W Xenon lamp	Nessler's reagent	[77]
16.	Ru_2.5_SiW_11_Fe@δ‐Bi_2_O_3_	Z‐Scheme	121	300 W Xenon lamp	Nessler's reagent	[90]
17.	1D/2D Bi_2_O_3_/Bi_2_MoO_6_	Oxygen vacancies + heterojunction	55.21	300 W Xenon lamp	Indophenol blue method	[109]
18.	Bi_2_O_3_@CoAl‐LDHs	Z‐scheme	48.7	300 W Xenon lamp, Na_2_SO_3_	Nessler's reagent	[110]
19.	Bi_2_O_3_/CdMoO_4_	S‐scheme	323	300 W Xenon lamp, 10% methanol	Nessler's reagent	[97]
20.	C@Bi_2_O_3_‐BiOCl‐Bi	Carbon supported heterostructure	400	300 W Xenon lamp	Nessler's reagent	[104]
21.	Ni‐incorporated ZrO_2_/Bi_2_O_3_	Oxygen vacancies + type‐II	9668.2	Sunlight, 5% TEOA	UV–vis spectrophotometer (Cary 60 UV–vis spectrophotometer)	[83]
22.	Sulfur‐vacancy‐rich Bi_2_S_3_ nanorods	Sulfur vacancies	51.04	300 W Xenon lamp	Nessler's reagent	[72]
23.	Bi/Bi_2_S_3_/SnS_2_	Sulfur vacancies + Z‐scheme	96.4	300 W Xenon lamp	Nessler's reagent	[111]
24.	Ag‐Bi_2_S_3_/MnOS	Z/p‐n heterojunction	2.97	Simulated solar light	Nessler's reagent	[112]
25.	Bi_2_S_3_/OV‐Bi_2_MoO_6_	S‐scheme + oxygen vacancies	126	Simulated solar light	Nessler's reagent	[96]
26.	Bi_2_S_3_/BiOBr	Heterojunction	878	300 W Xe lamp	Nessler's reagent	[113]
27.	p‐Cu_3_BiS_3_/n‐Bi_2_S_3_	Z‐scheme	66.38	Power density to 2.69 W cm^−2^	Indophenol blue method	[114]
28.	Bi_2_Te_3_/BiOCl	Heterojunction	315.9	Visible light	Nessler's reagent	[115]
29.	Bi_2_S_3_@PCN	Embedded in the host	3880	Visible light	Nessler's reagent	[103]
30.	TiO_2_QDs/Bi_2_O_3_/ NaBiS_2_	Tandem *n*‐*n*‐p heterojunctions	2902	500 W Xenon lamp	Nessler's reagent	[116]
31.	C‐Bi_2_S_3_‐MOF	Doping + Host	147	Sunlight	—	[117]
32.	Ce‐BiOBr/Bi_2_S_3_@LC	Embedded in the host + Z‐scheme	571.94	300 W Xe lamp	Nessler's reagent	[118]

## 5 Challenges and Current Limitations

Despite substantial progress in developing bismuth chalcogenides and related materials for photocatalytic nitrogen reduction, several fundamental and practical challenges continue to hinder their efficiency and real‐world applicability. A primary limitation arises from the low solubility of nitrogen in aqueous solutions (20 mg/L,20°C, 100 kPa), which limits the contact area and contact time between the photocatalyst and nitrogen molecules. Second, the intrinsic inertness of molecular nitrogen, whose strong N≡N triple bond requires effective adsorption and activation on the catalyst surface. Although bismuth‐based materials exhibit favorable electronic characteristics, including potential π back‐donation interactions, nitrogen chemisorption under ambient conditions often remains weak due to insufficient active sites and limited orbital overlap. Third, in aqueous photocatalytic systems, the HER competes directly with nitrogen reduction, as photogenerated electrons tend to preferentially reduce protons rather than N_2_, thereby compromising ammonia selectivity.

Another significant challenge is rapid electron–hole recombination, which limits the availability of charge carriers for surface redox reactions. Even with strategies such as heterojunction construction, defect engineering, elemental doping, and morphology control, maintaining long‐lived charge separation and efficient interfacial electron transfer remains difficult. In addition, precise band‐edge alignment is essential to provide adequate driving force for N_2_ reduction while minimizing HER; however, many pristine bismuth chalcogenides possess suboptimal CB positions. Limited surface area and low density of accessible active sites further constrain catalytic performance, while defect‐rich systems, though beneficial for N_2_ adsorption, may suffer from instability, photocorrosion, or structural reconstruction during prolonged irradiation.

Beyond material‐related issues, reliable performance evaluation, false‐positive NH_3_ detection, and reproducibility pose major bottlenecks in photocatalytic NRR research. The typically low ammonia production rates are highly susceptible to contamination from external nitrogen sources, reagents, or catalyst residues. Although various quantification techniques, including spectrophotometry, IC, ion‐selective electrodes, NMR analysis, and fluorometric methods, are employed, inconsistencies between analytical approaches and insufficient use of isotopic (^15^N_2_) validation often led to questionable reproducibility. Furthermore, the absence of standardized testing protocols, variations in light sources and reactor configurations, and differences in experimental conditions make cross‐comparison of reported results challenging. Finally, most investigations remain at laboratory scale, and critical issues such as long‐term stability, catalyst recyclability, photon utilization efficiency, and scalability toward practical photocatalytic ammonia production systems remain largely unresolved.

## 6 Future Directions

Developing environmentally benign, energy‐efficient technologies for artificial ammonia synthesis is essential to reducing CO_2_ emissions and addressing global energy and environmental challenges. Among the various bismuth chalcogenides investigated for photocatalytic nitrogen reduction, Bi_2_S_3_ and Bi_2_O_3_ have emerged as the most promising candidates owing to their suitable band structures, strong visible‐light absorption, tunable defect chemistry, and versatility in heterostructure construction. Comparative analysis of reported studies indicates that heterojunction engineering, particularly Z‐scheme and S‐scheme architectures, offers the greatest potential for achieving high NRR by simultaneously promoting charge separation, preserving strong redox activity, and facilitating N_2_ activation. Defect engineering, especially the introduction of sulfur or OVs, has also proven highly effective in creating active sites for N_2_ adsorption and weakening the N≡N bond. Furthermore, benchmark performance comparisons suggest that integrating multiple modification strategies, such as defect‐rich heterojunctions, doped Z‐scheme systems, and porous hybrid architectures, generally produces the highest photocatalytic activities owing to synergistic improvements in charge transport, light harvesting, and nitrogen activation.

Looking forward, future research should focus on precise electronic‐structure engineering to optimize band‐edge alignment, enhance π back‐donation to adsorbed N_2_ molecules, and suppress the competing HER. Advanced defect‐modulation strategies, including controlled vacancy generation, atomic‐level doping, single‐atom modification, facet engineering, and strain tuning, may provide new opportunities to improve nitrogen activation efficiency and catalytic stability. The rational design of Type‐II, Z‐scheme, and S‐scheme heterojunctions should be further refined to maximize charge separation while maintaining strong reduction and oxidation potentials. In addition, integrating bismuth chalcogenides with porous frameworks, conductive matrices, and hybrid host structures is expected to enhance surface accessibility, mass transport, and photon utilization efficiency. Coupling photocatalytic systems with photoelectrochemical (PEC) configurations represents another promising direction, as external bias can facilitate charge separation, improve reaction selectivity, and suppress competing HER.

Equally important is the development of a deeper mechanistic understanding of photocatalytic NRR through advanced in situ and operando spectroscopic techniques combined with theoretical calculations. These approaches can help identify true active sites, reaction intermediates, and rate‐limiting steps, thereby enabling rational catalyst design. Future studies should also prioritize sacrificial‐agent‐free systems, rigorous ^15^N_2_ isotope validation, contamination‐free reaction protocols, and cross‐validated ammonia quantification methods to ensure data reliability and reproducibility. Furthermore, reporting standard metrics such as apparent quantum efficiency, solar‐to‐ammonia conversion efficiency, and long‐term operational stability will facilitate meaningful benchmarking across different systems. Ultimately, bridging the gap between laboratory‐scale demonstrations and scalable solar‐driven ammonia production remains the key challenge and opportunity for translating bismuth chalcogenide‐based photocatalysts into practical NRR technologies.

## 7 Conclusion

Bismuth chalcogenides and related materials have emerged as highly promising visible‐light‐responsive photocatalysts for sustainable nitrogen reduction to ammonia under ambient conditions. Their tunable electronic structures, strong visible‐light absorption, surface plasmon resonance effects, and favorable band‐edge positions provide unique advantages for efficient solar energy harvesting and N_2_ activation. Significant progress has been achieved through defect engineering, elemental doping, heterojunction formation, and hybrid structure design, which collectively enhance charge separation, light utilization, and catalytic activity. Among these strategies, heterojunction engineering and defect modulation have demonstrated particularly strong potential for promoting N_2_ adsorption, activation, and selective reduction. Despite these advances, challenges including inefficient N_2_ activation, competing hydrogen evolution, charge‐carrier recombination, limited active‐site density, and concerns about the reliability and reproducibility of NH_3_ quantification remain major obstacles to practical implementation. Future efforts should focus on integrating multiple catalyst‐engineering strategies, developing sacrificial‐agent‐free systems, employing advanced in situ/operando characterization techniques, and establishing rigorous experimental validation protocols. Addressing these limitations through rational material design, advanced mechanistic understanding, and standardized evaluation methodologies will be crucial for advancing the field. With continued interdisciplinary efforts, bismuth chalcogenide‐based photocatalysts hold significant potential for advancing green, sustainable ammonia production technologies.

## Author Contributions

**Mukesh K. Verma**: conceptualization, data curation, formal analysis, investigation, writing – original draft, and writing – review and editing. **Muhammad D Bala**: conceptualization, supervision, and writing – review and editing. **Phindile B. Khoza**: conceptualization, supervision, and writing – review and editing. **Tshephiso R. Papo**: conceptualization, supervision, and writing – review and editing. **Mzamo L. Shozi**: conceptualization, supervision, writing – review and editing, and project administration.

## Conflicts of Interest

The authors declare no conflicts of interest.

## Data Availability

The authors have nothing to report.
